# The antiseizure medication stiripentol inhibits mitochondrial complex I by blocking early-stage electron transfer

**DOI:** 10.1016/j.jbc.2026.111179

**Published:** 2026-01-20

**Authors:** Cyrielle L. Bouchez, Thibaut Molinié, Guillaume Duranthon, Sebastian Lillo, Corinne Pellon, Nadia El Mammeri, Benjamin Dartigues, Philippe Pasdois, Benoit Pinson, Macha Nikolski, Anne Devin, Arnaud Mourier, Roland T. Ullrich, Thomas Daubon

**Affiliations:** 1 Departement I of Internal Medicine, University Hospital Cologne, Cologne, Germany; 2 Translational Research for Infectious Diseases and Oncology Center Research, University of Cologne, Cologne, Germany; 3 University of Cologne, Faculty of Medicine and University Hospital Cologne, Mildred Scheel of Oncology, Germany; 4 Cologne Excellence Cluster on Cellular Stress Responses in Aging-Associated Diseases (CECAD), Cologne, Germany; 5 Université de Bordeaux, CNRS UMR5095, IBGC, Bordeaux, France; 6 Université de Bordeaux, CNRS, Bordeaux INP, CBMN, Pessac, France; 7 University Bordeaux, Bordeaux Bioinformatics Center (CBiB), Bordeaux, France; 8 Université de Bordeaux, TBMCore Service Analyses Metaboliques CNRS UAR 3427 INSERM US05, Bordeaux, France; 9 Center for Integrated Oncology Aachen/Bonn/Cologne/Dusseldorf, University Hospital Cologne, Cologne, Germany

**Keywords:** cancer, mitochondria, complex I, respiration, antiepileptic

## Abstract

The oxidation of NADH is essential for maintaining cellular redox balance and supporting cell metabolism. Mitochondrial complex I (NADH:ubiquinone oxidoreductase) plays a central role in this process by coupling NADH oxidation to electron transfer and proton translocation across the inner mitochondrial membrane. We previously reported that the antiseizure medication stiripentol decreases lactate production and mitochondrial respiration, suggesting an impact on NADH turnover beyond its known inhibition of lactate dehydrogenase. In this study, we identify complex I as a target of stiripentol across multiple species and cell types. Biochemical and spectroscopic analyses demonstrate that stiripentol inhibits NADH oxidation and electron transfer through a mechanism distinct from that of classical ubiquinone pocket inhibitors such as rotenone or piericidin A. Remarkably, stiripentol acts upstream of the ubiquinone reduction site, representing the first example of a complex I inhibitor with a binding site within the N-module. These findings uncover a previously unrecognized mode of complex I inhibition and link stiripentol’s metabolic effects to direct modulation of mitochondrial NADH oxidation. This work broadens the understanding of stiripentol’s mechanism of action and highlights its potential to modulate redox metabolism in cancer cells.

Mitochondria are essential organelles for the production of energy in the cell but also allow the formation of many metabolic intermediates. Mitochondria are involved in ATP synthesis, which primarily relies on the oxidative phosphorylation system (OXPHOS). This system is based on a chemiosmotic coupling between the respiratory chain and a phosphorylating system. The respiratory chain itself is composed of several complexes through which electrons are transferred from reduced cofactors and metabolites to the final acceptor, oxygen. The two main pathways that fuel the respiratory chain are complex I/NADH-coenzyme Q reductase, or complex II/succinate dehydrogenase.

The reoxidation of NADH is a critical process for cell fate and viability, and therefore can be achieved through independent processes such as fermentation and respiration involving key players like lactate dehydrogenase (LDH) and complex I. The complex I of the respiratory chain is composed of a hydrophilic domain with seven iron-sulfur centers and an FMN-type flavin group essential for the NADH oxidase-coenzyme Q reductase activity (N-module), the quinone binding site (Q-module) and a hydrophobic domain forming the proton-pumping part (P-module) (1, 2, 3). According to the literature, complex I inhibitors, such as rotenone and Piericidin A, bind specifically to this ubiquinone reduction pocket or Q-module (4, 5). To date, only a few inhibitors of complex I were characterized to target another action site (6, 7, 8).

Stiripentol (Diacomit) is an antiseizure medication (ASM) primarily used in the context of the Dravet Syndrome to treat epileptic paediatric patients (9, 10, 11). Stiripentol belongs to the group of aromatic allyl alcohols and may potentiate the effect of other ASM through pharmacokinetic interactions. It increases the levels of gamma-aminobutyric acid (GABA), a major inhibitory neurotransmitter that regulates electrical activity in the central nervous system by different mechanisms of action (12, 13, 14). Stiripentol inhibits GABA transporters, thereby preventing its reuptake, and also blocks the activity of GABA transaminase, the primary enzyme responsible for GABA degradation (12, 13, 14).

In recent years, several studies have shown that stiripentol also has the property of inhibiting lactate dehydrogenase (LDH) activity (15, 16, 17, 18). LDHs are tetrameric enzymes composed of two types of subunits, LDHA and LDHB. These reversible enzymes couple the reduction of pyruvate to lactate to the oxidation of NADH. In their study, Sada *et al*. found that stiripentol inhibits LDH in a non-competitive manner, which suggests that stiripentol does not physically interfere with the enzymatic active sites of lactate or pyruvate (15). Besides, in our previous study we showed that stiripentol could impact the respiratory rate, suggesting a potential interaction with the respiratory chain (16).

In this study, we demonstrate that stiripentol is a molecule that is able to inhibit complex I of the respiratory chain across different cell lines and species. We also characterize its unique mechanism of action, which differs from that of currently known inhibitors. We here describe for the first time that a complex I inhibitor can act independent of the ubiquinone reduction pocket. This work contributes to the understanding of the action mode of stiripentol as an ASM and anti-cancer drug and describes a pluripotent role in controlling energy metabolism.

## Results

### Stiripentol impacts tumor cell proliferation rate and viability

**Figure 1:**
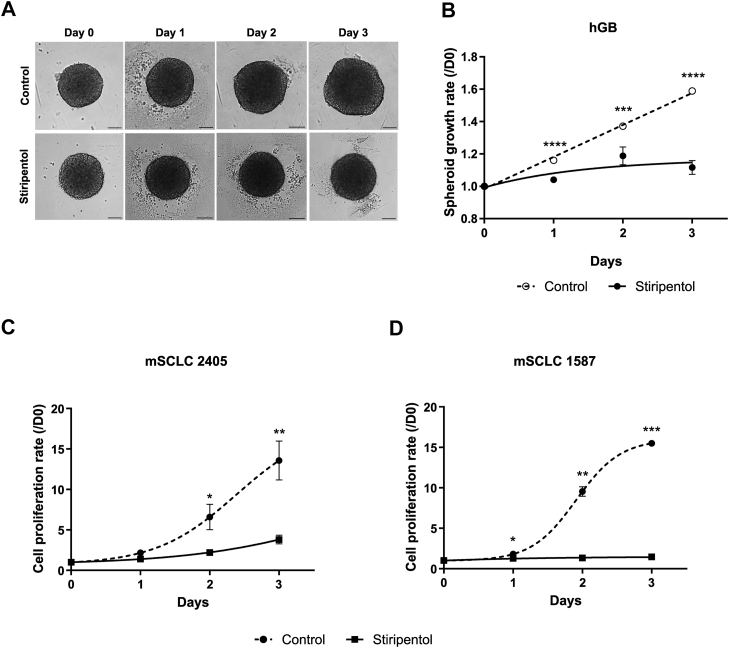
**Impact of Stiripentol on the growth rate**. *A*, area of P3 spheroid is measured every day for 3 days during incubation with or without 500 μM stiripentol. Data are represented as mean ± SEM (n = 3). Scale bar: 100 μm. (8–10 spheroids per condition, n = 3). *B*, P3 spheroid growth recorded over 3 days during incubation with or without 500 μM stiripentol. Data are represented as mean ± SEM (n = 3). (8–10 spheroids per condition, n = 3). *C*, mSCLC 2405 growth rate measured on cultures with or without 250 μM stiripentol. Growth rates for each condition were calculated in each experiment from counting of cells in cultures each day for a period of 3 days. Data are represented as mean ± SEM (n = 6). *D*, mSCLC 1578 growth rate measured on cultures with or without 250 μM stiripentol. Growth rates for each condition were calculated in each experiment from counting of cells in cultures each day for 3 days. Data are represented as mean ± SEM (n = 6). for all experiments: ∗, *p* < 0.05; ∗∗, *p* < 0.01; ∗∗∗, *p* < 0.001; ∗∗∗∗, *p* < 0.0001.

Previous work on the human glioblastoma (hGB) stem-like cell model showed that stiripentol treatment reduced the spheroid growth and invasiveness (16). To determine the potential of stiripentol as anticancer treatment, we performed growth rate and cell viability assays in hGB cells and in two other cancer cell lines of small cell lung cancer (SCLC) derived from a primary SCLC tumor (mSCLC 2405) or from liver metastasis of SCLC (mSCLC 1578). Our results reproduced the ones previously described but with a higher efficiency from day 1 after treatment (Fig. 1*A* and Fig. S1*A*). Control and stiripentol-treated spheroid growths were then quantified and showed that stiripentol treatment at 500 μM reduced the spheroid growth rate by 1.5-fold at day 3 (Fig. 1*B*). We then measured the proliferation and viability of mouse SCLC cells (mSCLC 2405 and mSCLC 1578) with different concentrations of stiripentol (Fig. S1*B*). For both cell lines, a treatment at a concentration of 500 μM stiripentol induced an increase in cell death from 30% to 50% (Fig. S1*B*). Moreover, a treatment of 250 μM with stiripentol reduced the proliferation rate in the primary SCLC (mSCLC-2405) as well as in the liver metastatic cells (mSCLC-1578), as observed for hGB cells (Fig. 1, *C* and *D*), without impacting the viability (Fig. S1*B*).

All of these results showed that stiripentol impacts the proliferation rate and viability of human and mouse tumor cell lines.

### Stiripentol impacts respiratory capacity and reshuffles ubiquinone oxidoreductase quantity

**Figure 2:**
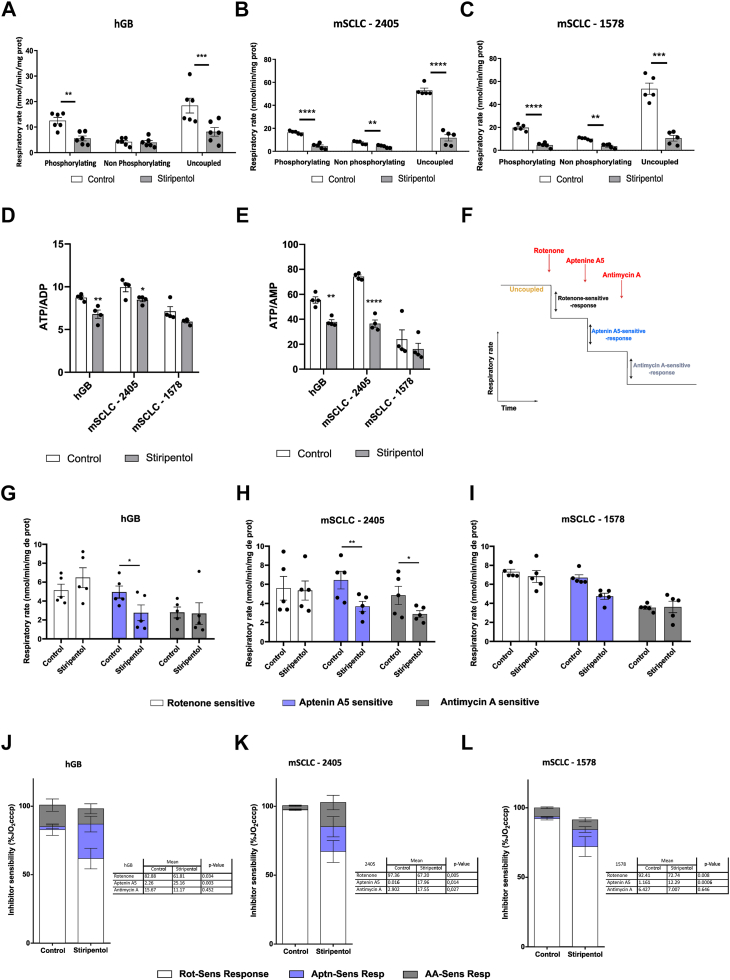
**Impact of long-term Stiripentol treatment on reorganisation of respiratory chain complexes**. *A–C*, respiration of glioblastoma P3 cells treated with or without 500 μM stiripentol (*A*) and of mSCLC 2405 or 1578 cells treated with or without 250 μM stiripentol (*B* and *C*) for 3 days assessed under different respiratory states (phosphorylating, non-phosphorylating, and uncoupled). Data are represented as mean ± SEM (n = 6). for all experiments: ∗∗, *p* < 0.01; ∗∗∗, *p* < 0.001; ∗∗∗∗, *p* < 0.0001. *D*, ATP/ADP ratio. *E*, ATP/AMP ratio on hGB, mSCLC 2405 and mSCLC 1578 with or without 500 μM stiripentol (for hGB) and 250 μM Stiripentol (for mSCLC) were calculated from the individual cells data used to calculate the mean values in Table 1. Statistics were carried out as described in Table 1, for all: ∗, *p* < 0.05; ∗∗, *p* < 0.01; ∗∗∗. *F*, illustration of the experimental setup for the measurement of the NADH and succinate contribution. *G–I*, Rotenone- (*G*), Aptenin A5- (*H*) and Antimycin A- (*I*) sensitive respiration of glioblastoma P3 cells treated with or without 500 μM stiripentol and of mSCLC 2405 or 1578 cells treated with or without 250 μM stiripentol for 3 days assessed under uncoupled conditions and after a successive addition of the indicated inhibitors. Data are represented as mean ± SEM (n = 5). for all experiments: ∗, *p* < 0.05; ∗∗, *p* < 0.01; ∗∗∗. *J–L*, respective contribution of NADH and succinate dehydrogenases to the uncoupled respiration cultured in NBM-glucose (for human cells) (*J*) or in RPMI 1640 (for murine cells) (*K* and *L*) medium after a treatment with 500 μM or 250 μM stiripentol for 3 days. Data are represented as mean ± SEM (n = 4 or 6). For all experiments, the mean and *p*-value are summarized in the table besides each graph.

We previously showed that stiripentol decreases hGB cell respiratory capacities (16). To elucidate how stiripentol modifies such OXPHOS capacities, we studied the respiratory rate of the different cell lines after 3 days of treatment. The oxygen consumption under phosphorylating (in the presence of ADP and functional ATP synthase), non-phosphorylating (with an inhibitor of ATP synthase), and uncoupled conditions (maximal respiratory chain capacity) was measured using high-resolution O2K oxygraphs. In hGB cells cultured in glucose-complemented medium, the respiratory rate decreased under phosphorylating and uncoupled conditions upon stiripentol treatment at 500 μM, by 50 to 80% compared to the control (Fig. 2*A*). In murine SCLC cell lines, respiration measured under the same conditions was reduced to 50% of the respiration assessed in non-treated cells after a treatment with 250 μM of stiripentol (Fig. 2, *B* and *C*). In parallel, we measured the ATP/ADP and ATP/AMP ratios with or without stiripentol treatment to assess its role in the maintenance of energy metabolism linked to the adenosine phosphate. Our data showed that stiripentol treatment triggers a significant decrease in this ratio for hGB and mSCLC 2405, with a similar tendency for mSCLC 1578 (Fig. 2, *D* and *E*, and Table 1). A decrease of ATP/ADP and ATP/AMP ratio was also observed after other OXPHOS inhibitors, use like rotenone (19), antimycin A (20) or oligomycin (21). These results corroborate a long-term inhibition of OXPHOS and a rewiring of metabolism.

As we used an uncoupling molecule, we hypothesize that stiripentol has an impact on the respiratory chain activity independently of a potential effect on the phosphorylating system. In parallel, we tried to determine the sensitivity of the mouse cell lines to stiripentol by following the inhibition of the respiratory rate at different concentrations of stiripentol. The respiratory rate was inhibited by approximately 70% for each cell line with 100 μM of stiripentol and by 90% with 200 μM of stiripentol (Fig. S2, *A*–*B*). With 500 μM of stiripentol, the maximum of inhibition was reached compared to the inhibition under antimycin A, a specific complex III inhibitor (Fig. S2, *A*–*B*).

To understand the mode of action of stiripentol on the respiratory chain and to decipher if stiripentol directly targets OXPHOS complex activity in a cellular context, we studied the respective contribution of complex I and complex II by successively inhibiting these complexes (complex I with rotenone) complex II with aptenin A5, and complex III with antimycin A) (Fig. 2*F*), as described before (22, 23). Antimycin A was used here to verify the activity of all the other potential ubiquinone oxidoreductases, which can provide electrons to the respiratory chain (22, 23). The sensitivity to rotenone was decreased while the sensitivity to aptenin A5 was increased in the three cell lines (Fig. 2, *G*–*I*), suggesting a modification of the contribution of complexes I and II to mitochondrial respiration. To decipher the contribution of each complex for the global respiration, we then calculated the repartition of the respiration rate after adding the different inhibitors under uncoupling conditions for each cell lines (Fig. 2, *J*–*L*). For the three cell lines, the repartition of respiration dependent on the different complexes showed a decrease of rotenone sensibility by 20 to 30%. In parallel, the sensibility to aptenin A5 increased by 10 to 20% in the total respiration rate. These results confirmed a decrease of the complex I contribution on the total respiratory chain activity while the complex II contribution increased. We noticed, for the mSCLC 2405, an increase in the antimycin A sensibility (Fig. 2, *H* and *K*).

### Stiripentol affects complex I stability

**Figure 3:**
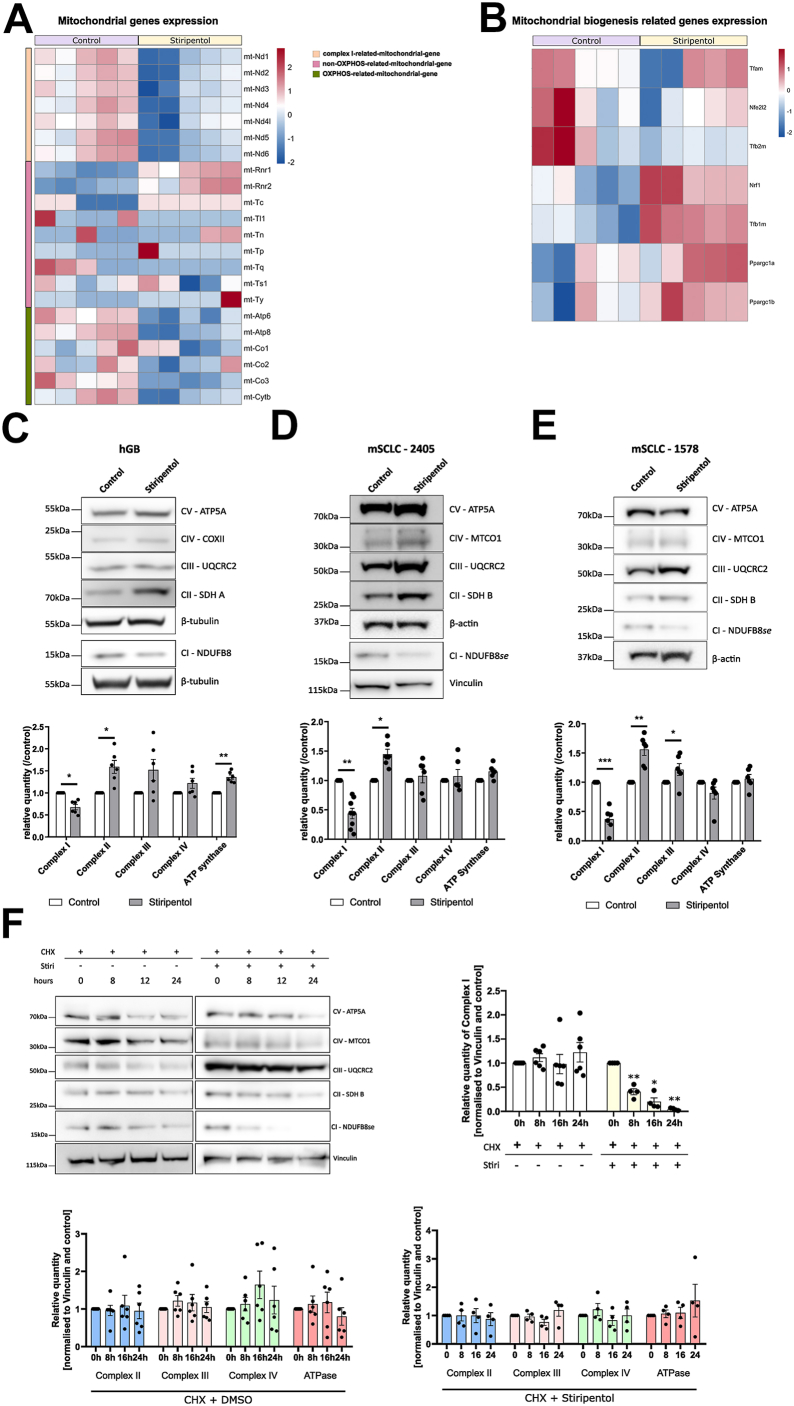
**Stiripentol impacts the complex I expression and its stability in time**. *A*, heat map of RNA sequencing for mtGenes in mSCLC 2405 with or without 250 μM stiripentol (n = 5). *B*, heat map of RNA sequencing for nuclear genes encoding for mitochondrial biogenesis factors in mSCLC 2405 with or without 250 μM stiripentol (n = 5). Full RNA sequencing data are available in Table 2. *C*, *up*: the relative levels of OXPHOS complexes were determined in control P3 cells and treated cells with 500 μM stiripentol. Total protein extracts were analyzed by SDS-PAGE and Western blotting using antibodies directed against different subunits of OXPHOS complex and Tubulin as a loading control. *Bottom*: Results representative of three experiments are shown. One subunit for each complex signal was quantified. Signal intensity was normalized to Tubulin signal and expressed as a percentage of control for each complex and each experiment. The bar graph shows the mean percentage of control ± SEM (n = 4). ∗, *p* < 0.05; ∗∗, *p* < 0.01. *D* and *E*, *up*: the relative levels of OXPHOS complexes were determined in control mSCLC 2405 or 1578 cells and treated cells with 250 μM stiripentol. Total protein extracts were analyzed by SDS-PAGE and Western blotting using antibodies directed against different subunits of OXPHOS complex and Actin as a loading control. *Bottom*: Results representative of three experiments are shown. One subunit for each complex signal was quantified. Signal intensity was normalized to Actin signal and expressed as a percentage of control for each complex and each experiment. The bar graph shows the mean percentage of control ± SEM (n = 6). for all experiments: ∗, *p* < 0.05; ∗∗, *p* < 0.01; ∗∗∗, *p* < 0.001. *F*, *left*: the relative levels of OXPHOS complex were determined in control mSCLC 2405 cells and treated cells with 250 μM stiripentol in presence of cycloheximide. Total protein extracts were analyzed by SDS-PAGE and Western blotting using antibodies directed against different subunits of OXPHOS complex and Vinculin as a loading control. *right*: results representative for complex I of at least four experiments are shown. *Bottom*: results representative for the other complexes of at least four experiments are shown. One subunit for each complex signal was quantified. Signal intensity was normalized to Vinculin signal and expressed as a percentage of control for each complex and each experiment. The bar graph shows the mean percentage of control ± SEM (n = 4).

To understand how complex I activity decreases after stiripentol treatment, we examined the expression of the different OXPHOS complexes. The expression of nuclear genes coding for complex I or other OXPHOS complexes did not decrease significantly after stiripentol treatment (Fig. S3*A*). However, the mitochondrial genes coding for complex I significantly decreased after treatment (Fig. 3*A* and Fig. S3*B*). Additionally, the expression of other mitochondrial genes related to the other OXPHOS complexes slightly decreased (Fig. 3*A* and Fig. S3*B* and Table 2). Subsequently, we demonstrated that the expression of the other mitochondrial genes, described in the Figure S4, or the expression of nuclear genes encoding factors involved in mitochondrial biogenesis did not change following stiripentol treatment (Fig. 3, *A* and *B*), suggesting that the OXPHOS deficiency is not linked to a decrease in mitochondrial content. Thus, stiripentol mainly impacts the expression of complex I-related mitochondrial genes.

We next evaluated the OXPHOS complex subunit relative content by Western blot with or without stiripentol treatment in each cell line. Our data consistently showed that, for all three cell lines, a reduction of the expression of the complex I subunit NDUFB8, while complex II subunit SDHA increased after stiripentol treatment (Fig. 3, *C*–*E*), followed by a shift in respiration from complex I to complex II (Fig. 2, *G*–*L*).

The observed decrease in complex I content may be attributed to either reduced transcriptional levels or an elevated turnover rate of the complex. We determined the stability of the complex *via* a cycloheximide experiment. After treatment with cycloheximide, an inhibitor of protein synthesis, stiripentol triggered a decrease in complex I over time, while being stable in the control condition (Fig. 3*F*). The stability of the other OXPHOS complexes was not impacted by stiripentol (Fig. 3*F*).

Taken together, these findings indicate that long-term stiripentol treatment reduces respiratory rates by specifically targeting complex I expression and activity. This effect may trigger a compensatory redistribution of ubiquinone oxidoreductase activity toward complex II.

### Stiripentol instantaneously inhibits cell respiration

**Figure 4:**
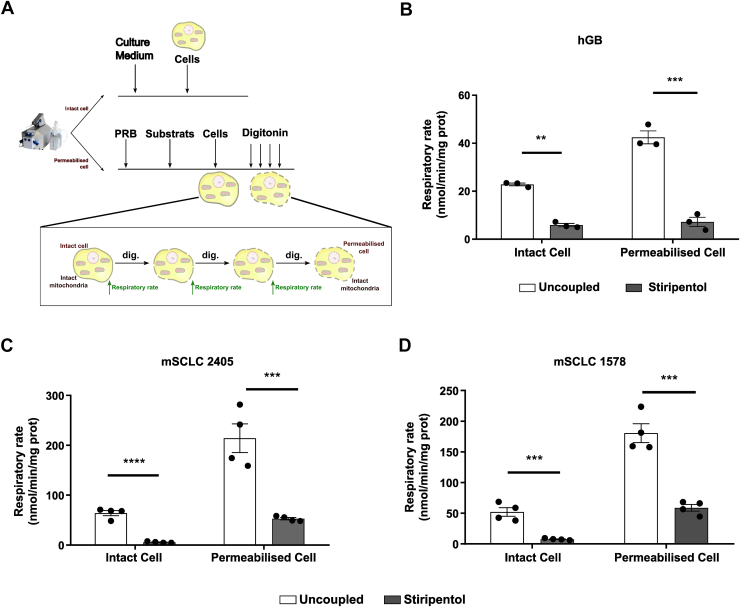
**Observation of instantaneous inhibition of respiration with stiripentol on intact and permeabilised cells**. *A*, illustration of the experimental setup of the use of digitonin (dig.). *B*, respiration of glioblastoma P3 cells assessed under uncoupled respiratory cultured in NBM-glucose and measure in NBM-glucose to the intact cells and measure in phosphate buffer with Succinate, Pyruvate, Glutamate, Malate to the permeabilised cells with or without stiripentol addition. Data are represented as mean ± SEM (n = 3). for all experiments: ∗∗, *p* < 0.01; ∗∗∗, *p* < 0.001. *C* and *D*, respiration of mSCLC 2405 or 1578 cells assessed under uncoupled respiratory cultured in RPMI 1640 medium and measure in RPMI 1640 medium to the intact cells and measure in phosphate buffer with Succinate, Pyruvate, Glutamate, Malate to the permeabilised cells with or without stiripentol addition. Data are represented as mean ± SEM (n = 4). for all experiments: ∗∗∗, *p* < 0.001; ∗∗∗∗, *p* < 0.0001.

Our results showed that stiripentol long-term treatment affects respiratory capacity *via* a regulation of complex I expression and activity, notably by affecting transcriptional expression and its stability. Thus, to elucidate whether the effect of the stiripentol on the respiratory chain is only observed during long-term treatment, which would suggest a signaling/regulatory role for stiripentol rather than a direct effect on the respiratory chain, we investigated the effect of stiripentol on the respiratory rate in a short time after stiripentol treatment. We measured the respiratory rate of intact and digitonin-permeabilized cells respectively incubated with glucose and a non-limiting concentration of pyruvate, glutamate and malate to selectively fuel complex I. Next, we assessed the oxygen consumption on intact and permeabilized cells after instantaneous stiripentol treatment using high-resolution O2K oxygraphs under uncoupling conditions for the three cell types and followed the permeabilization by respiration measurement. Permeabilization of the plasma membrane by the digitonin, until a plateau in respiration (Fig. S5), allows to choose the metabolite to provide electron to the respiratory chain at the desired concentration to avoid any limitation of substrate or inhibitor diffusion (Fig. 4*A*). We observed in hGB cells, in mSCLC 2405 and in mSCLC 1578 that the inhibition of the respiratory rate was observed immediately after injecting 500 μM of stiripentol (Fig. 4, *B*–*D*). Moreover, as the inhibition is similar after permeabilization of the plasma membrane, it showed that stiripentol can diffuse through the mitochondrial membrane and acts on the respiratory chain at the maximum of its capacity at this concentration without membrane constraint (Fig. 4, *B*–*D*).

Taken together these results show that stiripentol is able to inhibit instantaneously the respiratory chain activity, as observed after a long-term treatment. Besides its role in the expression and stability of complex I, we thus assumed that stiripentol also targets the pathways that provide NADH to the complex I or directly the complex I.

### Stiripentol is a complex I inhibitor

**Figure 5:**
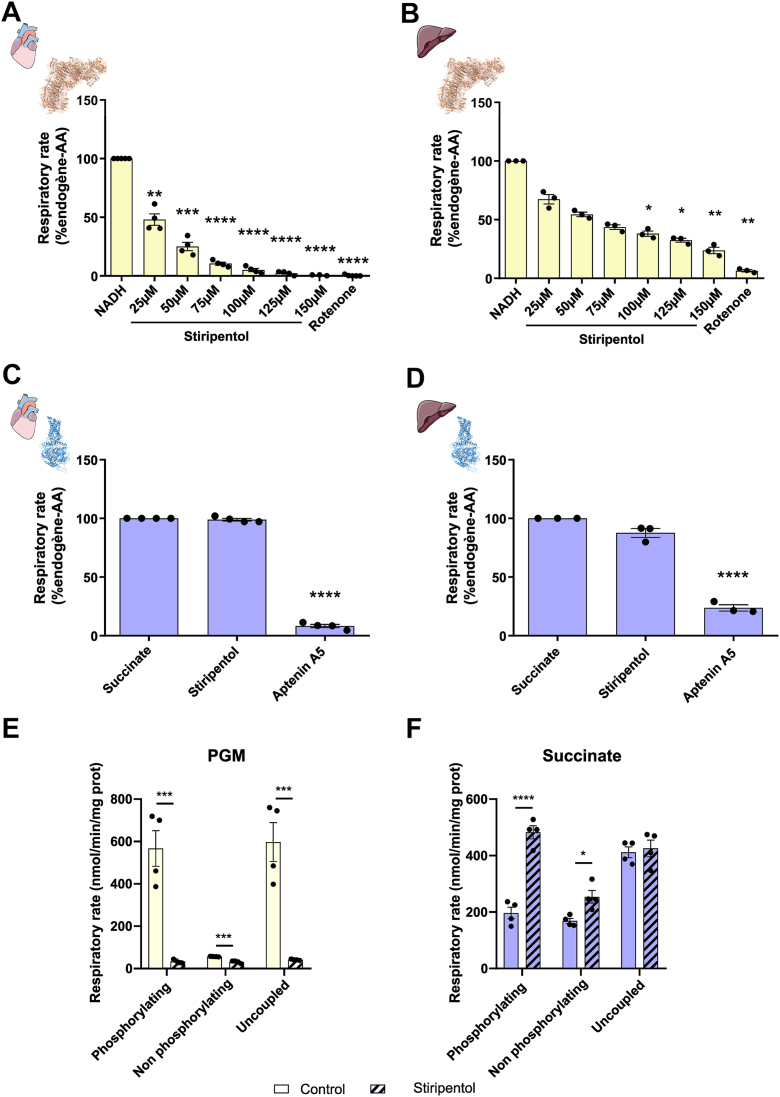
**Stiripentol is an inhibitor of Complex I**. *A*, oxygen consumption assessed with permeabilised heart mitochondrial fed with NADH and increased concentrations of stiripentol and a final addition of 20 nM rotenone. Results are expressed as a percentage of NADH condition for each e xperiment. The bar graph shows the mean percentage of NADH condition ± SEM (n = 4). *B*, oxygen consumption assessed with permeabilised liver mitochondrial fed with NADH and increased concentrations of stiripentol and a final addition of 20 nM rotenone. Results are expressed as a percentage of NADH condition for each experiment. The bar graph shows the mean percentage of NADH condition ± SEM (n = 3). *C*, oxygen consumption assessed with permeabilised heart mitochondrial fed with succinate and an addition of 200 mM stiripentol and a final addition of 20 nM aptenin A5. Results are expressed as a percentage of succinate condition for each experiment. The bar graph shows the mean percentage of succinate condition ± SEM (n = 4). ∗∗∗∗, *p* < 0.0001. *D*, oxygen consumption assessed with permeabilised liver mitochondrial fed with succinate and an addition of 200 mM stiripentol and a final addition of 20 nM aptenin A5. Results are expressed as a percentage of succinate condition for each experiment. The bar graph shows the mean percentage of succinate condition ± SEM (n = 3). ∗∗∗∗, *p* < 0.0001. *E*, oxygen consumption assessed with intact heart mitochondrial fed with pyruvate, glutamate and malate (PGM) in phosphorylating conditions after an incubation with DMSO (control) or stiripentol for 15 min. The bar graph shows the mean ± SEM (n = 4). *F*, oxygen consumption assessed with intact heart mitochondrial fed with succinate in phosphorylating conditions after an incubation with DMSO (control) or stiripentol for 15 min. The bar graph shows the mean ± SEM (n = 4).

To determine if the respiratory rate decrease due to stiripentol treatment is linked to a specific inhibition of the complex I and not an impairment of the NADH supply, we measured respiratory rate on permeabilized mitochondria from heart and liver, which present a strong difference in their NADH and succinate oxidative capacities (23). Permeabilized mitochondria offer the advantage to study the respiratory chain capacity independently of the internal metabolism or metabolite transporters. Mitochondria were fed with NADH as complex I substrate, or succinate as complex II substrate. When the respiratory rate was only driven by complex I activity, stiripentol was able to inhibit the oxygen consumption (Fig. 5, *A* and *B*). However, stiripentol was not able to inhibit the respiratory rate when it was only driven by complex II activity (Fig. 5, *C* and *D*). To further evaluate the impact of stiripentol on complex I activity, we also tested its effect on mitochondrial metabolism. Rotenone boosts complex II respiration by blocking complex I, which prevents oxaloacetate buildup—a natural inhibitor of complex II. This removes the inhibition, allowing complex II to work at full capacity. (23). To do so, we incubated heart mitochondria in the presence of complex I substrates (pyruvate, glutamate malate) or complex II substrate (succinate). These data showed that complex I respiration was inhibited by stiripentol in intact mitochondria (Fig. 5*E*). However, they also showed that succinate respiration was increased after stiripentol treatment (Fig. 5*F*), as previously reported for rotenone treatment (23).

These data show that stiripentol inhibits the respiratory rate by preventing NADH reoxidation in the matrix *and* reducing oxaloacetate accumulation. Unlike rotenone, stiripentol does not directly or specifically inhibit complex I.

### Stiripentol has a different binding site than rotenone

**Figure 6:**
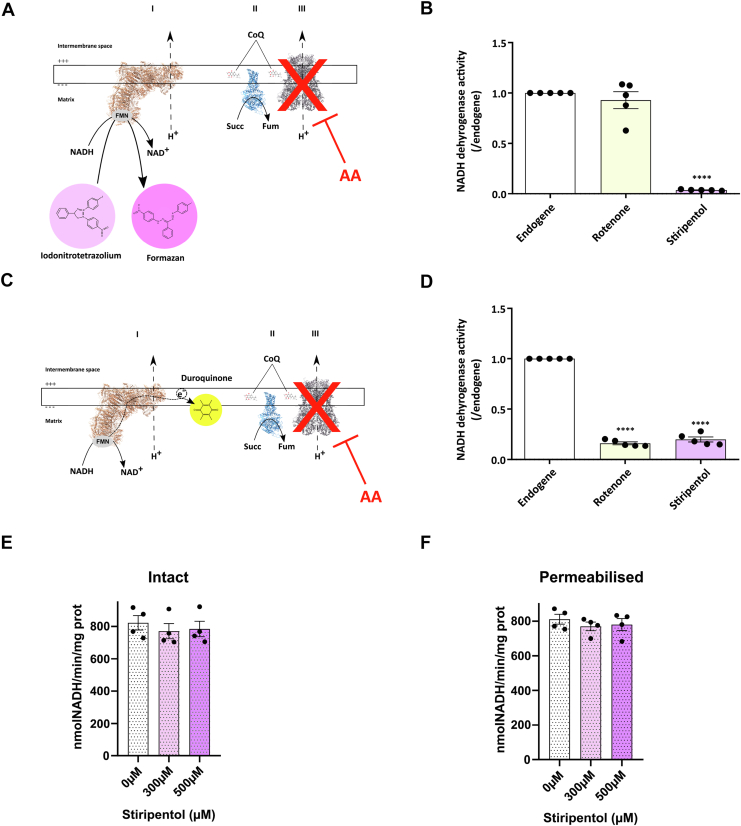
**Inhibitory effect of stiripentol seems to be upstream of quinone pocket localization**. *A*, scheme of INT mechanism at level of complex I. *B*, NADH dehydrogenase activity measured on permeabilised heart mitochondria in spectrophotometry in presence of antimycin A, NADH and INT and then treated with rotenone (20 nM) or stiripentol (150 μM). Results are expressed as a percentage of NADH condition for each experiment. The bar graph shows the mean percentage of NADH condition ± SEM (n = 4). *C*, scheme of duroquinone mechanism at level of complex I. *D*, Complex I activity measured on permeabilised heart mitochondria in spectrophotometry in presence of antimycin A, NADH and duroquinone and then treated with rotenone (20 nM) or stiripentol (150 μM). Results are expressed as a percentage of NADH condition for each experiment. The bar graph shows the mean percentage of NADH condition ± SEM (n = 4). for all experiments: ∗∗∗∗, *p* < 0.0001. *E* and *F*, NADH dehydrogenases activity assessed with intact (*E*) or permeabilised (*F*) *S*. *cerevisiae* mitochondria fed with NADH and increased concentrations of stiripentol. The bar graph shows the mean ± SEM (n = 4).

To go further and due to the inhibitory activity of stiripentol on LDHs (16, 17, 18), we studied the potential binding site of stiripentol on complex I. We measured complex I activity *via* two different spectrophotometric approaches. First, mitochondria were incubated with iodonitrotetrazelium (INT) and NADH to measure complex I activity. INT plays the role of electron acceptor, and its product (formazan) can be spectrophotometrically followed at 500 nm (Fig. 6*A*). Our results showed that only stiripentol, and not rotenone, was able to inhibit the formazan production (Fig. 6*B*). Then, to prove that the entire complex I can be inhibited by these two inhibitors, mitochondria were incubated with NADH and duroquinone, a soluble quinone, as a final electron acceptor (Fig. 6*C*). In these conditions, the NADH oxidation is catalysed by complex I, and the NADH consumption can be followed at 340 nm. By using duroquinone, this approach allows for driving the electron flow from NADH oxidation to the coenzyme Q binding pocket and by extension to determine the entire complex I activity. Our results showed that rotenone and stiripentol were both able to inhibit the complex I activity with this approach (Fig. 6*D*). These results demonstrate that the reduction of INT is upstream of the coenzyme Q reduction site and that rotenone and stiripentol do not have the same action site to inhibit the complex I.

Our data showed that the site of action of stiripentol is not localized within the NADH active site. To further characterize the mode of action of stiripentol, we performed NADH activity measurement on an isolated NADH dehydrogenase from *Saccharomyces cerevisiae*. In *S*. *cerevisiae*, the respiratory chain does not have complex I but instead presents an internal (Ndi) and an external (Nde) NADH coenzyme Q oxidoreductases (Nd systems) that do not couple electron flow to proton translocation (Fig. S6*A*). These Nd systems are insensitive to classical complex I inhibitors such as rotenone or piericidin A (24, 25). Our data showed that stiripentol did not inhibit the Nde or Ndi activity (Fig. 6, *E* and *F*). Thus, the site of action is probably downstream of the NADH binding site. Interestingly, we compared the sensitivity to stiripentol with the OXPHOS system from *Candida utilis*. The respiratory chain of *C*. *utilis* possesses an Nde and a proton-pumping complex I (Fig. S6*B*), which are respectively resistant and sensitive to classical complex I inhibitors used for mammalian complex I (25). In our experiment, mitochondria were only incubated with NADH as a substrate of the NADH dehydrogenase. Our results showed that stiripentol was able to inhibit the respiration in *C*. *utilis* (Fig. S6*C*), unlike in *S*. *cerevisiae*, indicating that stiripentol can inhibit complex I across different species.

These results showed that stiripentol is an inhibitor of complex I with a new action site, in opposition to all the other classical complex I inhibitors. In particular, stiripentol does not target the ubiquinone reduction pocket but more probably inhibits the N-module of the complex I.

### The predicted binding site of stiripentol differs from the rotenone binding site

We investigated the binding site of stiripentol on mitochondrial complex I by performing blind docking with CB-DOCK2, using both the active (PDB 6G2J) and an inactive form of the enzyme (PDB 6G72) states. Rotenone, a well-characterized inhibitor, was used as a reference. For each ligand and structural state, we examined the two top-scoring poses and identified interacting residues according to the original chain assignments in the deposited PDBs.

**Figure 7:**
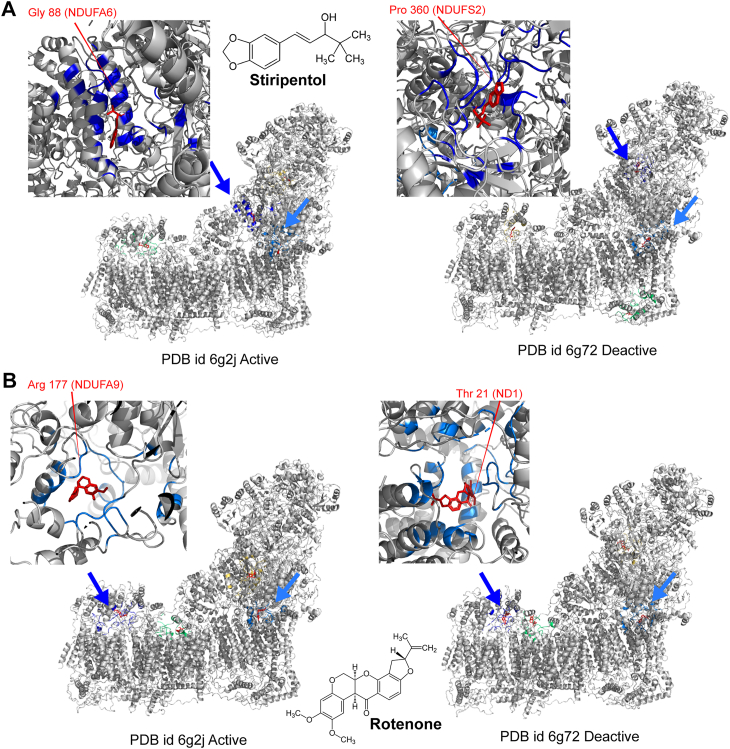
**Predicted binding poses of stiripentol and rotenone**. Predicted binding poses of stiripentol (*A*) and rotenone (*B*) in the active (PDB 6G2J) and deactive (PDB 6G72) states of mitochondrial complex I, as obtained from CB-DOCK2 blind docking. For each ligand, the two highest-scoring poses (rank one and rank 2) are shown. Rotenone consistently docks near the ubiquinone-binding channel (rank 2) in both conformational states, in agreement with experimental evidence, whereas stiripentol favors more peripheral, matrix-exposed interfaces (rank 1) with only secondary access to the Q-path vestibule (rank 2). These results highlight the distinct binding site preferences of the two ligands and suggest an allosteric mode of action for stiripentol compared to the canonical inhibitory mechanism of rotenone. Table 3 is a list of ligand-contact residues in human Complex I across four conditions as predicted by CB-Dock2.

For stiripentol in the active state, the highest-ranked pose (−8.3 kcal/mol) was located at a matrix-facing interface involving residues from NDUFS3 (chain C, positions 158–189) and a broad surface of the accessory subunit NDUFA6 (chain W, positions 31–114), with contributions from the ACP subunit (chain T). This region lies outside the canonical ubiquinone pathway and suggests an allosteric N/Q interface site. A second pose (−7.5 kcal/mol) contacted ND3 (chain A), ND1 (chain H), and NDUFA9 (chain P, residues 25–327), placing the compound near the vestibule of the ubiquinone channel but with weaker predicted affinity. In the deactive state, stiripentol again favored a matrix-side N/Q interface formed by NDUFS3 (chain C), NDUFS2 (chain D), NDUFS4 (chain Q), and NDUFA5 (chain V), while a second pose (−7.6 kcal/mol) engaged NDUFS7 (chain B) together with NDUFS2 (chain D) and extensive contacts from ND1 (chain H), which define the inner junction of the Q pathway. Taken together, these results suggest that stiripentol does not strongly occupy the canonical Q site but instead shows a recurring preference for peripheral interfaces, indicating multiple shallow binding possibilities (Fig. 7*A*, and Table 3).

Rotenone showed a clearer and more consistent binding pattern. In the active state, the second-ranked pose (−9.3 kcal/mol) corresponded to the canonical ubiquinone channel, with interactions involving ND3 (chain A), ND1 (chain H), NDUFS7 (chain B), and NDUFA9 (chain P). The top-ranked pose (−9.6 kcal/mol) was instead localized to a hydrophobic cleft at the ND4–ND5 interface (chains L and M with NDUFB subunits), which is likely a decoy site. The same pattern was observed in the deactive state: the best-scoring pose (−10.1 kcal/mol) was found in the ND4/ND5 cleft, while the second pose (−9.4 kcal/mol) occupied the NDUFS7–NDUFS2–ND1 junction, the expected rotenone site. Since experimental data strongly support rotenone binding within the ubiquinone channel, we interpret the second-ranked poses as biologically relevant and the top-ranked poses as docking artifacts (Fig. 7*B*, and Table 3).

In summary, stiripentol appears to favor peripheral binding interfaces, whereas rotenone consistently targets the ubiquinone channel, in line with its known mechanism of inhibition. Taken together, these results suggest a fundamental difference between the site of action of stiripentol and rotenone.

### Stiripentol has a different mechanism of action as compared with rotenone

The action site of stiripentol being different from rotenone, we wondered if these differences can also impact other parameters linked to complex I inhibition.

**Figure 8:**
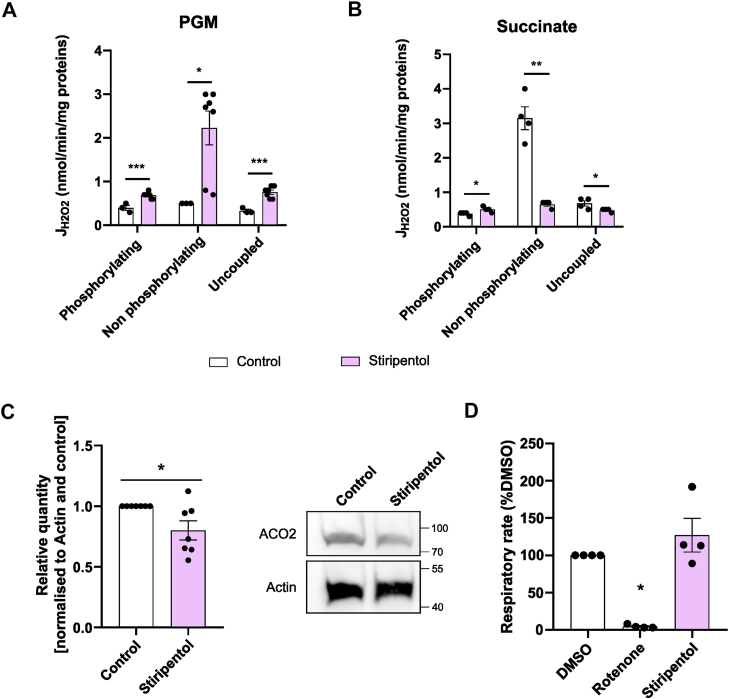
**Stiripentol and rotenone have different mechanisms of action**. *A*, ROS production rate assessed with intact heart mitochondrial fed with pyruvate, glutamate and malate (PGM) in phosphorylating conditions after an incubation with DMSO (control) or stiripentol for 15 min. The bar graph shows the mean percentage of NADH condition ± SEM (n = 4). *B*, ROS production rate assessed with intact heart mitochondrial fed with succinate in phosphorylating conditions after an incubation with DMSO (control) or stiripentol for 15 min. The bar graph shows the mean percentage of NADH condition ± SEM (n = 4). *C*, *left*: the relative levels of ACO2 were determined in control mSCLC 2405 cells and treated cells with 250 μM stiripentol in presence of cycloheximide. Total protein extracts were analyzed by SDS-PAGE and Western blotting using antibodies directed against ACO2 and Actin as a loading control. *right*: representative images of Western blot. Signal intensity was normalized to Actin signal and expressed as a percentage of control each experiment. The bar graph shows the mean percentage of control ± SEM (n = 4). *D*, oxygen consumption assessed with intact heart mitochondrial fed with pyruvate, glutamate, malate and succinate in a phosphorylating condition after an incubation with DMSO (control) or stiripentol for 15 min and a cycle of washing. The bar graph shows the mean percentage of DMSO condition ± SEM (n = 4).

One of the characteristics of rotenone action is its stimulating effect on ROS production while the complex I is fed in substrates (26, 27) contrasting with its inhibitory effect on the production of ROS in a context of reverse electron flow (28, 29, 30). Subsequently, we studied the ROS production in isolated heart mitochondria and cells. Heart mitochondria fueled with pyruvate, glutamate, malate and succinate in a phosphorylating condition show that stiripentol significantly increased ROS production when acutely added (Fig. 8*A*). Furthermore, in the case of reverse electron flow (succinate in non-phosphorylating conditions), stiripentol, like rotenone (28, 29, 31), abolishes ROS production, meaning that reverse electron flow from succinate oxidation was also blocked by stiripentol (Fig. 8*B*). Two ROS production sites are known in complex I: one in the FMN pocket and one in the quinone pocket. Thus, we hypothesized that stiripentol action site might be between these two sites, which fit with the binding site predicted (Fig. 7). To validate the increase in ROS production in a cellular model, we performed Western blot targeting ACO2, which is affected by ROS production (31, 32, 33). Our data showed that after a stiripentol treatment, ACO2 is slightly but significantly decreased (Fig. 8*C*). This result confirms that stiripentol induces an increase in ROS production as reported for rotenone (31, 32, 33).

Next, we studied the reversibility of the stiripentol inhibition. We incubated the mitochondria with stiripentol for 15 min. Our results showed that at this time is efficient for a complete inhibition of complex I (Fig. 3). We washed the mitochondria to remove all the unbound stiripentol, then we performed respiratory measurement. In parallel, we reproduce this experiment with rotenone, known to have an irreversible binding for the complex I (34, 35). The results showed that stiripentol was not able to inhibit the respiratory rate after washing, contrary to rotenone, suggesting that stiripentol is a reversible inhibitor, for short-term incubation (Fig. 8*D*).

### Stiripentol cannot inhibit all mammalian NADH dehydrogenases

**Figure 9:**
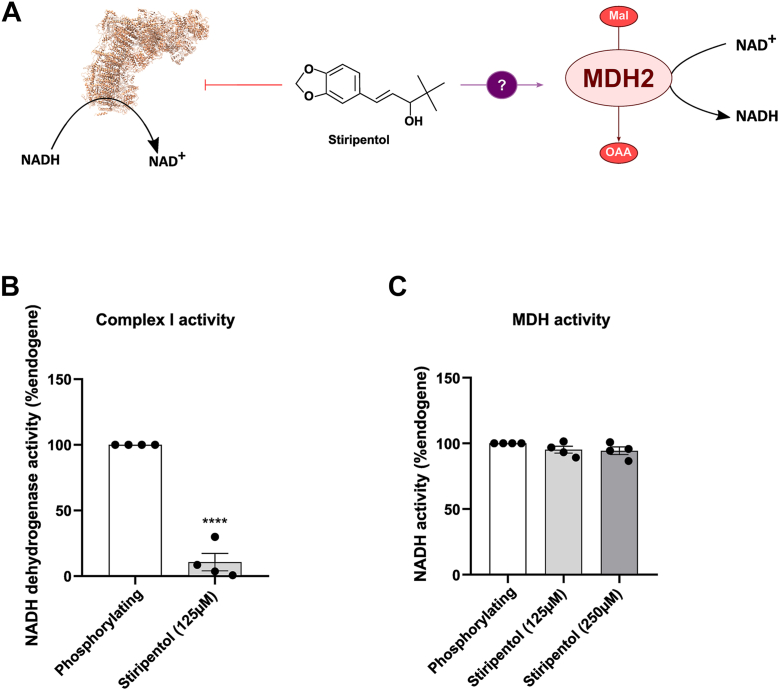
**Stiripentol has no effect on malate dehydrogenase 2**. *A*, scheme of the potential inhibition of MDH2 *via* stiripentol. *B*, complex I activity measured on permeabilised heart mitochondria in spectrophotometry in the presence of antimycin A, NADH and duroquinone. Results are expressed as a percentage of the NADH condition for each experiment. The bar graph shows the mean percentage of endogenous condition ± SEM (n = 4). ∗∗∗∗, *p* < 0.0001. *C*, MDH activity measured on permeabilized heart mitochondria in spectrophotometry in the presence of malate, NAD^+^ and Acetyl CoA. Results are expressed as a percentage of endogene condition for each experiment. The bar graph shows the mean percentage of NADH condition ± SEM (n = 4).

Stiripentol has been shown to inhibit LDH (15, 16), and we showed in this study that it also inhibits mammalian respiratory complex I, we were wondering if this molecule can target all the NADH dehydrogenases in mammalian cells such as malate dehydrogenases (MDHs), which is a prominent source of NADH fueling complex I in cardiac mitochondria (Fig. 9*A*) (23). To study the impact of stiripentol on MDH2, we incubated isolated heart permeabilized mitochondrial with MDH substrate, NAD^+^ and malate, and we measured by monitoring spectrophotometrically NADH production. In parallel, complex I inhibition was controlled in the same conditions in the presence of NADH and duroquinone as previously described. Our data showed that stiripentol inhibited complex I (Fig. 9*B*) but was not able to inhibit the MDH2 activity even at high concentration (Fig. 9*C*). Taken together, these data show that stiripentol can selectively inhibit several types of NADH dehydrogenase along evolutional kingdom.

## Discussion

In this study, we deciphered a new function for stiripentol as a respiratory chain complex I inhibitor. We performed our experiments in several models, hGB cells, mSCLC and yeast. We used different concentrations for mammalian cells: 500 μM for human cells and 250 μM for mouse cell lines. For hGB cells, we previously showed that the spheroid size difference between the untreated condition and the stiripentol condition was linked to a growth rate decrease and not to a decrease in cell viability (16). As hGB cells grow in spheroids, they are more resistant to the cytotoxicity effect of the stiripentol.

Unexpectedly, we demonstrated that stiripentol, besides its described role as a lactate dehydrogenase inhibitor, can inhibit multiple NADH dehydrogenases, including those from different species, but not all of them. These data suggest the possibility of a shared and specific binding site recognition by stiripentol, which is conserved across evolution in some NADH dehydrogenases. We notably demonstrated the inhibition of complex I by stiripentol *via* respiration measurement. These results were obtained instantaneously after stiripentol injection and thus show that stiripentol affects the mouse and human respiratory chain directly, independent of a potential proteome reshuffling. The mode of action of rotenone as an inhibitor of complex I of the respiratory chain is well documented. Rotenone specifically binds to the ubiquinone reduction pocket, preventing the transfer of electrons from the iron-sulfur cluster N2 to the coenzyme Q (4, 36). Nevertheless, stiripentol appears to act through a distinct mechanism.

In parallel, we also demonstrated that after a long-term stiripentol inhibited complex I-dependent respiratory rate was inhibited, but also enhanced complex II-dependent respiratory rate. We could also show that a part of the oxygen consumption flux sensitive to the antimycin A was increased in some cell lines. This remaining respiratory rate, dependent on complex III inhibition, but not dependent on complex I nor complex II, might be associated with the activity of other coenzyme Q oxidoreductases that can sustain the respiratory chain in electrons, such as dihydroorotate dehydrogenase (DHODH), Electron-transferring flavoprotein (ETF) or mitochondrial glycerol-3-phosphate dehydrogenase (mG3PDH) (37, 38, 39, 40). This rewiring of the electron entrance in the respiration chain suggests that the cells try to compensate for the decrease of complex I contribution in the feeding of the respiratory chain, although this is not sufficient to reach a normal respiratory capacity.

Our study suggests that stiripentol has a different action site than the other known complex I inhibitor, notably rotenone. Rotenone is consistently predicted to bind within the ubiquinone channel in both active and deactive conformations, in agreement with structural and biochemical data. Stiripentol, however, shows no consistent docking to this channel; instead, it repeatedly favors peripheral, matrix-side interfaces at the N/Q module junction, with only secondary access to the Q-path vestibule. This supports the hypothesis that stiripentol may act through an allosteric mechanism rather than direct competition with ubiquinone. Importantly, these findings are based solely on CB-DOCK2 predictions, which do not account for membrane context, conformational flexibility, or solvent effects. Thus, the results should be viewed as testable hypotheses, requiring experimental validation through complementary experimental approaches, such as site-directed mutagenesis, binding assays, or structural studies.

This also raises the possibility of a regulatory domain that can impact complex I activity and its turnover since stiripentol induces a decrease in complex I expression and stability, and the expression of the complex I subunits after long-term treatment, a phenomenon not observed with rotenone. The different mechanism of action and the decrease in complex I activity over time suggest that stiripentol binding immediately affects the activity of complex I and, over time, its stability, whereas an inhibition by rotenone has no consequence on the complex relative quantity (data not shown). In regard to the decrease in the complex I-related-mitochondrial-gene expression, we did not study the stiripentol mechanism of action; thus, we cannot conclude if this decrease is a consequence of the inhibition of complex I or not. Our experiments do not allow us to conclude that rotenone does not affect the stability of complex I, unlike stiripentol. We hypothesize that stiripentol and rotenone activate distinct ROS production sites, which could account for their differential effects on complex I stability.

Additionally, our results demonstrate that stiripentol directly inhibits complex I, reducing respiration without disrupting the mitochondrial NADH supply *via* MDH2.

Stiripentol is an ASM used primarily in the treatment of Dravet syndrome, a rare and severe form of epilepsy. It modulates the activity of ion channels in the brain, helping to reduce neuronal excitability and prevent epileptic seizures. Additionally, studies have suggested that stiripentol may have potential in the treatment of certain forms of cancer (16, 17, 41, 42, 43). In particular, we have investigated its role in glioblastoma in relation to its function as an inhibitor of LDH, another NADH dehydrogenase. Our preliminary data described here that the stiripentol can also be used for other types of cancer, such as SCLC.

Our results show that stiripentol can inhibit the respiratory chain activity after a few minutes, as well as after 3 days of *in vitro* treatment. Moreover, we demonstrated that this inhibition specifically affects the activity of complex I and, surprisingly, allows a reshuffling of the respiratory chain, with a decrease in the relative expression of complex I and an increase in complex II. This result is associated with a decrease in complex-dependent respiration and an increase in the complex II dependent-respiration and other coenzyme Q oxidoreductases to sustain the respiratory chain. This result indicates an increase in the activity of these dehydrogenases to compensate for the inhibition of complex I, inducing a rewiring of the cancer metabolism. However, this compensation is not sufficient to restore the oxygen consumption capacity observed before treatment with stiripentol. To survive, cells need to maintain their redox metabolism to support the catabolic and anabolic pathways, signaling, or ROS protection pathway, which require NAD(P) entities to recycle glutathione, for example. In mammals, there are two major pathways to maintain the NAD^+^/NADH ratio: lactic fermentation and complex I of the mitochondria. By inhibiting both lactate dehydrogenase and complex I, stiripentol seems to be a promising complementary strategy against cancer proliferation, especially in contexts where cancer cells can adapt and switch from a glycolytic metabolism to an OXPHOS metabolism, such as in a Warburg effect context. By affecting the overall redox metabolism, stiripentol may prevent the adaptation that allows some types of cancer to survive anti-cancer treatment.

## Experimental procedures

### Cell culture

Patient-derived GBM P3 cells were cultured in NeuroBasal medium (NBM) supplemented with B27, heparin (100 U/μl), 20 ng/ml basic FGF and Penicillin – Streptomycin (1000 U/ml) at 37 °C in 5% CO_2_ incubator. Stable cell lines were generated by infecting with lentiviral particles. The genetically engineered SCLC mouse model is driven by conditional deletion of the tumor suppressor genes Rb1 and Tp53. The mice received anesthesia by intraperitoneal injection of xylazine (Bayer AG)/ketamine (Zoetis Inc.) (10/100 mg/kg/KGW; max. injection volume 0,1 ml/10g mice), then underwent an intratracheal Adeno-Cre virus application. The University of Iowa Viral Vector Core (http://www.medicine.uiowa.edu/vectorcore) provided the viral vectors. Tumor development was monitored by μCT (LaTheta mCT, Hitachi Alcoa Medical, Ltd). For murine SCLC cell lines, lung tumors or liver metastasis of the autochthonous mouse model were mechanically dissociated using 40 μm cell strainers (BD Falcon) and ACK lysis buffer. Isolated cells were cultivated in RPMI medium (Life Technologies) with 10% FBS (Sigma-Aldrich) and 1% antibiotics (penicillin/streptomycin, Life Technologies) and frequently screened for *mycoplasma* contamination by PCR. After five passages, the cell lines were defined as stable, and they were used for functional experiments.

### Spheroid experimental assays

Protocols are described in detail in a previous article (44). Briefly, P3 spheroids of uniform size were prepared from 10^4^ dissociated cells in 100 μl NBM with 0.4% methylcellulose in a 96-well round-bottom plate. The spheroids were used after 3 days of formation. For spheroid growth, in each well, 100 μl NBM were added with or without 500 μM stiripentol.

### Western blot

Cells were washed twice in PBS. Cells were lysed in RIPA buffer (10 mM Tris-HCl, pH 7.4, 150 mM NaCl, 0.5% NP-40, 1%TritonX-100, 1 mM EDTA) containing protease and phosphatase inhibitors. Protein concentrations were determined using *DC* protein assay (BioRad). Cell lysates were resuspended in Laemmli Buffer (62.5 mM Tris, 10% glycerol, 2.5% SDS, 5% β-mercaptoethanol, pH 6.8). The protein extracts were separated by SDS-PAGE in 4% to 12% precasted gels (Invitrogen) performed according to the method of Laemmli (45). After electro-transfer onto nitrocellulose membranes (Amersham Biosciences), proteins were probed with the desired primary antibodies: α-OXPHOS (Abcam for human cells #ab110411 and mouse cell line #ab110413); α-Tubulin (Merck T5168) and detected using peroxidase-conjugated secondary antibodies (Jackson ImmunoResearch) and ECL Prime reagent (BioRad), according to the manufacturers’ instructions or using IR-Dye 800 labelled secondary antibodies. Colorimetric bands were recorded with Optical Densitometry of an Amersham ImageQuant 800 for hGB cells and with the UVP ChemStudio PLUS Imagers of an Analytik Jena for all the other blots. Signal quantifications were done using the ImageJ software.

### Heart and liver mitochondria

Isolation of heart and liver mitochondria was performed by differential centrifugation as previously described (46, 47). Briefly, right after the sacrifice, tissues were collected, minced and cleaned with mitochondria isolation buffer (MIB; 310 mM sucrose, 20 mM Tris-Base, 1 mM EGTA, pH 7.2) for the heart and MIB + BSA (MIB and 0.25 mg/ml of delipidated Bovine serum albumin (BSA)) for the liver. The pieces of heart were collected and homogenized with a few strokes of a Potter S homogenizer (Sartorius) using a loose Teflon pestle, in an ice cold MIB supplemented with trypsin (0.5 g/l). Liver tissues homogenization was performed in a Potter S homogenizer (Sartorius) using a Teflon pestle, and in ice-cold MIB + BSA. Mitochondria were isolated by differential centrifugation, a low-speed one (1,000g, 10 min, 4 °C) in a swing-out rotor and another one at high speed (3,500g, 10 min, 4 °C for heart mitochondria and 10,000g, 10 min, 4 °C for liver mitochondria) using a fixed-angle rotor. The pellet of crude mitochondria obtained is suspended in MIB + BSA. Protein concentration was measured using the Bio-Rad DC Protein Assay kit according to the provider’s instructions.

### Respiratory rate

#### Intact and permeabilized cells

Endogenous cell respiration was measured polarographically at 37 °C using a Clark-type electrode from Hansatech Instruments (Norfolk, United Kingdom). Substrate-driven respiration was assayed in digitonin-permeabilized cultured cells as reported (48). Briefly, trypsinized cells were washed with permeabilized-cell respiration buffer (PRB) containing 0.3 M mannitol, 10 mM KCl, 5 mM MgCl_2_, 0.5 mM EDTA, 0.5 mM EGTA, 1 mg/ml BSA and 10 mM KH_3_PO_4_ (pH 7.4). The cells were resuspended at ∼4∗10^6^ cells/ml in 1.5 ml of the same buffer air-equilibrated at 37 °C supplemented 10 units of hexokinase and 2 mM ADP. One ml of cell suspension was immediately placed into the polarographic chamber to measure endogenous respiration. High-resolution oxygen consumption rate of digitonin-permeabilized cells was performed as described previously (49) at 37 °C, using one million cells diluted in 2 ml of respiratory buffer (120 mM sucrose, 50 mM KCl, 20 mM Tris-Base, 4 mM KH2PO4, 2 mM MgCl2, 1 mM EDTA, pH 7.2) using an Oxygraph-2K (Oroboros). Briefly, cells were permeabilized with 0.02 mg/ml digitonin. The oxygen consumption rate under phosphorylating condition was assessed using either NADH-linked substrates (10 mM glutamate, 10 mM pyruvate and 5 mM malate) and 10 mM succinate or NADH only when cells were permeabilized, or 10 mM succinate in the presence of 2.5 mM ADP. From Figure 2, the oxygen consumption rate was measured using cells incubated in Neurobasal medium or RPMI 1640 medium and no substrate was added. The non-phosphorylating state was obtained after ATP synthesis inhibition using 0.75 mg/ml oligomycin. Mitochondrial respiration was uncoupled by successive addition of up to 0.4 mM CCCP to reach maximal oxygen consumption.

#### Permeabilized mammalian mitochondria

Activity of the respiratory chain was also recorded using permeabilized crude mitochondria obtained after freeze and thaw cycle. Oxygen consumption by the respiratory chain was measured as previously described (50), using 50 μg of heart and liver mitochondria diluted in 2 ml of potassium phosphate buffer (50 mM, pH 7.2) and incubated with saturating concentrations of cytochrome c (62.5 μg/ml), NADH (0.4 mM) and succinate (10 mM) in the presence of 2.5 mM ADP. No oxygen consumption could be detected in the absence of cytochrome c, and no oxygen consumption linked to complex I activity was recorded when permeabilized mitochondria were incubated with pyruvate, glutamate and malate, confirming the loss of integrity of the mitochondrial internal compartment. The determination of oxygen consumption link to electron coming from complex I and complex II was determined after the addition of rotenone (Rot, 20 nM) for complex I and/or atpenin A5 (Atpn, 20 nM) for complex II. As a control, antimycin A (AA, 100 nM) was added at the end of each experiment to control the proportion of oxygen consumption that is not linked to the respiratory chain.

#### Intact mammalian mitochondria

Heart-intact mitochondria were isolated by differential centrifugation from C57Bl/6N mouse genetic background as previously described (23). Oxygen consumption rate was measured using an Oxygraph-2K, and the H_2_O_2_ rate production using the Oroboros O2k-Fluo Smart-Module using the Smart Fluo-Sensors, green LEDs (GN), excitation peak of 525 nm at 37 °C, using 50 μg of heart mitochondria diluted in 2 ml of respiratory buffer (120 mM sucrose, 50 mM KCl, 20 mM Tris-Base, 4 mM KH_2_PO_4_, 2 mM MgCl_2_, 1 mM EDTA, pH 7.2) using an Oxygraph-2K (Oroboros). Oxygen consumption and H_2_O_2_ production rate was measured based on a previously described method (51) using Amplex Red (10 μM), horseradish peroxidase (1 U/ml), and SOD (1 μg/ml), with the different substrates combination: succinic acid (10 mM; pH 7.2), sodium pyruvate (10 mM; pH 7.2), L-Glutamate acid (5 mM; pH 7.2), L-Malic acid (5 mM; pH 7.2). Oxygen consumption rate was measured under three different conditions: in the phosphorylating state with addition of ADP (5 mM; pH 7.2), in the non-phosphorylating state with addition of oligomycin (50 ng/ml), and in the uncoupled state by successive addition of carbonyl cyanide m-chlorophenyl hydrazone (CCCP) up to 0.5 μM to reach the maximal respiration of the respiratory chain, and the respiration was ended by the addition of antimycin A (AA, 100 nM).

#### Permeabilized yeast mitochondria

Activity of the RC was also recorded using permeabilized yeast mitochondria. Oxygen consumption by the RC was measured as previously described (50), using 50 μg of *S*. *cerevisiae* and *C*. *utilis* mitochondria diluted in 2 ml of PRB) containing 0.65 M mannitol, 20 mM Tris, 1 mM EGTA, 1 mM magnesium acetate tetrahydrate and 10 mM KH_3_PO_4_ (pH 6.8). and incubated with saturating concentrations of cytochrome c (62.5 μg/ml), NADH (400 μM) in the presence of 2.5 mM ADP. As a control, antimycin A (AA, 100 nM) was added at the end of each experiment to control the proportion of oxygen consumption that is not linked to RC.

### RNA sequencing

Cells were seeded in a 6-well plate and exposed to different experimental conditions for 48 h. RNA was extracted using Qiagen RNeasy Mini Kit, according to the manufacturer's protocol (Qiagen). Corresponding to the manufacturer’s requirements, a concentration of 100 to 200 ng/μl was used from each sample. Libraries of 3′mRNA were obtained from total RNA using the Lexogen QuantSeq kit according to standard protocol. After validation and quantification (2200 TapeStation, Agilent Technologies and Qubit System, Invitrogen, respectively), pools of cDNA libraries were generated. Pools were quantified using the KAPA Library Quantification kit (Peqlab) and the 7900HT Sequence Detection System (Applied Biosystems, Foster City, PA, USA) and lastly sequenced on an Illumina HiSeq4000 or NovaSeq6000 sequencer using a 2 × 100 base pair protocol. A pre-analyze with Genavi is applied to normalize the results and obtain the count per million.

Volcano plots were generated in R using *ggplot2* and *ggrepel*. The x-axis shows log2 fold change (Stiripentol/Control) and the y-axis −log10(*p* value). Genes with *p* < 0.05 are shown as triangles; non-significant genes as dots. Mitochondrial and nuclear genes were annotated using predefined gene lists and colored by category; selected significant genes were labeled.

Heatmaps were generated from normalized expression matrices using *pheatmap*. Expression values were row-scaled (z-score). Columns represent five Control and five Stiripentol replicates. Genes were grouped by functional category, with row clustering disabled. Color scale indicates low to high expression (blue–white–red).

### Measurement of functionally isolated NADH dehydrogenase activity

NADH dehydrogenase activity was measured spectrophotometrically by monitoring the disappearance of NADH at 340 nm on either whole mitochondria (Ndep activity) or permeabilized mitochondria (Ndep and Ndip activity). The enzymes were functionally isolated by adding inhibitors of the respiratory chain downstream of the dehydrogenases (antimycin A - complex III inhibitor and KCN - complex IV inhibitor) and an artificial electron acceptor (Q2). Kinetics were performed by monitoring absorbance at 340 nm. Zero absorbance was set for 1 ml respiration buffer (0.65 M Mannitol, 10 mM Tris/maleate, 0.3 mM EGTA, 5 mM KH2PO4, pH 6.8) supplemented with 250 μM Q2, 0.1 μg antimycin A and 1 mM KCN, then 100 μM NADH were added. The reaction was started by the addition of 0.2 mg/ml mitochondria, and the NADH consumption rate was determined from the initial rate of the kinetics.

### NADH activity

NADH activity was also recorded using permeabilized crude mitochondria obtained after freeze and thaw cycle. Heart mitochondria were diluted in 2 ml of potassium phosphate buffer (50 mM, pH 7.2). Then, two protocols were established (1). NADH activity was assessed by using INT. Mitochondria were incubated with INT (0.05 mg/ml), NADH (400 μM), and antimycin A (100 nM). NADH activity was determined by monitoring spectrophotometrically (500 nm) the rate of appearance of formazan at 37 °C (2). NADH activity was assessed by using duroquinone. Mitochondria were incubated with duroquinone (100 μM), NADH (40 μM) and antimycin A (100 nM). NADH activity was determined by monitoring spectrophotometrically (340 nm) the rate of NAD^+^ formation. All activities (1 + 2) were challenged by the addition of rotenone (50 nM) or stiripentol (50 μM). The enzyme activity was calculated using an extinction coefficient of 6300 M^-1^ cm^-1^ at 340 nm for NADH.

### MDH activity

The mitochondrial complex I activity was assessed on permeabilized mitochondria (2 μg/ml) isolated from the mouse heart. The NADH dehydrogenase activity was assessed using a gold standard procedure in the presence of NADH (250 μM) and Duriquinone (100 μM) in a Tris (50 mM)/KCl (150 mM) buffer (pH 8) with antimycin A (0.2 μM). The NADH oxidation was followed spectrophotometrically at 340 nm. This activity was assessed in the absence or presence of stiripentol (125 μM).

The Malate dehydrogenase activity was assessed on permeabilized mitochondria isolated from the mouse heart. The Malate dehydrogenase activity was assessed spectrophotometrically at 340 nm in the presence of Malate (5 mM), NAD+ (300 μM) and Acetyl CoA (100 μM) in a Tris (50 mM)/KCl (150 mM) buffer (pH 8) with antimycin A (0.2 μM) and rotenone (4 nM). The effects of stiripentol were studied at 125 μM and 250 μM.

### Adenylic nucleotides quantification

ATP/ADP and ATP/AMP ratios were determined by measuring intracellular adenylic nucleotide content on hGB, mSCLC 2405 and mSCLC 1578 cells grown in the exponential proliferation phase and treated or not with stiripentol for $$$ hours (500 μM for hGB and 250 μM for mSCLC cells). Metabolites were extracted from cells using an ethanol boiling method, separated by ionic chromatography on an Integrion chromatography station and detected by their absorbance at 260 nm, as described in (52). The intracellular concentration of ATP, ADP, and AMP has been determined using standard curves obtained with pure compounds.

### Statistical analyses

Data are presented as mean ± SEM unless otherwise indicated in figure legends. Sample number (n) indicates the number of independent biological samples (individual mice) for each experiment. Sample numbers and experimental repeats are indicated in the figures. Statistical analysis was carried out using analysis of variance for all results. Prism software (GraphPad, San Diego, CA) was used for all tests. A *p* value of less than 0.05 was considered significant.

Two-tailed Mann-Whitney unpaired *t* test were used to calculate the statistical significance between two groups for all the results except for the growth rates and viability assay, where multiple *t* tests are performed to compare data to D0 or vehicle. We chose an interval of confidence at 95% and the *p*-value was considered statistically significant if inferior to 0.05. For the figures, ∗ = *p*-value < 0.05, ∗∗ = *p*-value < 0.01, ∗∗∗ = *p*-value < 0.001 and ∗∗∗∗ = *p*-value < 0.0001.

**Table 1:** Individual cells data used to calculate the mean values of ATP/ADP and ATP/AMP ratio

Conditions	[ATP]	[ADP]	[AMP]	ATP/ADP	ATP/AMP
	mM/cell	mM/cell	mM/cell		
hGB					
Control					
#1	2,22E-05	2,70E-06	4,48E-07	8,24	49,67
#2	2,19E-05	2,40E-06	3,53E-07	9,14	62,02
#3	2,21E-05	2,49E-06	4,01E-07	8,86	55,10
#4	2,21E-05	2,55E-06	4,01E-07	8,67	55,26
Stiripentol					
#1	1,24E-05	1,85E-06	3,51E-07	6,71	35,45
#2	1,27E-05	2,31E-06	3,51E-07	5,51	36,15
#3	1,18E-05	1,64E-06	2,73E-07	7,19	43,12
#4	1,00E-05	1,29E-06	2,73E-07	7,77	36,80
mSCLC-2405					
Control					
#1	2,25E-05	2,56E-06	1,22E-06	8,78	18,43
#2	2,38E-05	3,61E-06	1,63E-06	6,58	14,57
#3	2,56E-05	3,81E-06	1,60E-06	6,73	16,02
#4	2,57E-05	3,98E-06	5,50E-07	6,45	46,72
Stiripentol					
#1	2,46E-05	4,09E-06	8,29E-07	6,03	29,71
#2	2,22E-05	3,71E-06	2,30E-06	6,00	9,67
#3	2,02E-05	3,69E-06	1,73E-06	5,48	11,69
#4	2,00E-05	3,24E-06	1,49E-06	6,18	13,47
mSCLC-1578					
Control					
#1	2,14E-05	2,10E-06	2,96E-07	10,20	72,47
#2	1,97E-05	1,90E-06	2,60E-07	10,33	75,75
#3	2,18E-05	2,59E-06	3,05E-07	8,45	71,74
#4	2,07E-05	1,91E-06	2,69E-07	10,81	77,00
Stiripentol					
#1	4,17E-05	4,75E-06	1,32E-06	8,77	31,62
#2	4,04E-05	4,61E-06	1,11E-06	8,77	36,58
#3	4,48E-05	5,73E-06	9,99E-07	7,82	44,80
#4	4,38E-05	5,12E-06	1,32E-06	8,56	33,26

Concentrations of ATP, ADP and AMP per cells measured in hGB, mSCLC 2405 or 1578 cells with or without stiripentol treatment for 48 h.

**Table 2:** RNA sequencing of 2405 cells treated with stiripentol

Symbol	2405_Control_1	2405_Control_2	2405_Control_3	2405_Control_4	2405_Control_5	2405_Stiripentol_1	2405_Stiripentol_2	2405_Stiripentol_3	2405_Stiripentol_4	2405_Stiripentol_5	Mean Ctr	Mean Stiri	*t* test	*p* value	-Log *p*-value	q-value	log2FC	FC (stiri/ctr)	Difference (stiripentol-control)
mt-Atp6	855,771,814	803,253,101	776,432,399	81,847,299	81,083,423	666,442,645	630,280,605	717,387,433	641,332,504	746,748,118	812,952,907	680,438,261	000,088,643	0,000,886	305,256,628	0,003,283	−025,670,752	083,699,591	−1,325,146,457
mt-Atp8	840,764,242	800,242,368	855,726,518	885,817,559	889,856,047	628,415,106	615,804,012	708,236,752	720,313,161	768,020,934	854,481,347	688,157,993	000,103,122	0,001,031	298,674,133	0,003,283	−031,230,917	080,535,169	−1,663,233,539
mt-Co1	12,2,003,201	119,144,225	122,202,191	125,857,694	129,768,863	125,710,206	125,301,218	11,532,786	117,641,253	120,747,038	123,795,235	12,0,945,515	033,077,409	0,330,774	048,046,863	0,250,561	−003,359,851	097,698,038	−0,284,971,979
mt-Co2	82,251,933	781,615,228	785,318,895	840,379,556	814,777,247	771,506,843	745,901,322	781,115,845	787,376,755	834,983,152	808,922,051	784,176,784	02,145,307	0,214,531	066,850,994	017,106	−004,482,176	096,940,958	−0,247,452,676
mt-Co3	105,227,456	977,092,761	933,263,941	949,832,049	972,802,769	835,101,554	835,849,135	810,337,665	832,319,867	881,609,236	977,053,217	839,043,492	000,037,267	0,000,373	342,829,117	0,002,823	−021,969,155	085,874,902	−1,380,097,252
mt-Cytb	928,308,335	900,156,392	105,122,004	110,474,806	1,042,718	699,074,239	694,107,233	836,507,705	869,615,511	894,134,174	10,0,543,016	798,687,773	000,722,001	000,722	21,414,628	0,012,878	−033,210,935	079,437,419	−2,067,423,924
mt-Nd1	970,997,352	922,487,547	102,570,511	106,583,422	985,731,697	611,435,072	6,270,615	791,034,471	836,452,153	870,567,789	994,151,187	747,310,197	000,309,281	0,003,093	250,962,008	0,006,694	−041,175,806	075,170,679	−2,468,409,896
mt-Nd2	879,817,164	842,486,973	941,135,354	981,717,666	953,372,282	539,518,445	55,462,007	772,507,813	812,280,501	848,353,728	919,705,888	705,456,111	001,616,281	0,016,163	179,147,803	0,022,261	−038,261,625	076,704,534	−2,142,497,763
mt-Nd3	665,911,264	533,125,437	647,290,969	691,144,076	684,310,372	088,786,883	228,808,165	493,402,272	460,670,397	519,410,013	644,356,423	358,215,546	001,276,634	0,012,766	189,394,516	0,019,341	−084,703,098	055,592,764	−2,861,408,774
mt-Nd4	910,860,664	893,369,979	102,462,504	108,435,177	102,974,032	605,550,378	595,921,744	840,022,059	880,535,896	899,423,572	988,589,555	76,429,073	001,940,717	0,019,407	171,204,159	0,024,502	−037,125,013	077,311,229	−2,242,988,251
mt-Nd4l	639,618,499	592,428,891	621,217,762	690,002,953	629,074,467	291,710,517	210,932,077	518,720,486	569,532,191	584,485,889	634,468,515	435,076,232	003,472,588	0,034,726	145,934,524	0,040,469	−054,428,037	068,573,337	−1,993,922,825
mt-Nd5	896,108,589	867,424,299	970,123,283	101,415,511	102,331,929	709,164,517	70,913,294	814,373,336	843,961,786	873,551,489	954,226,116	790,036,813	000,765,041	000,765	211,633,856	0,012,878	−027,241,129	08,279,346	−1,641,893,024
mt-Nd6	713,580,028	679,274,357	773,952,058	810,285,132	817,004,289	444,038,883	459,395,085	579,613,315	607,155,173	637,019,712	758,819,173	545,444,434	000,209,875	0,002,099	267,798,756	0,005,299	−04,763,239	071,880,687	−2,133,747,389
mt-Rnr1	658,071,581	610,563,079	538,901,398	550,583,041	583,959,984	822,308,782	781,821,132	881,807,787	914,842,216	928,573,186	588,415,817	865,870,621	4,8652E-05	0,000,049	430,980,392	0,000,737	055,731,545	147,152,846	277,454,804
mt-Rnr2	722,097,135	702,335,069	730,055,388	754,256,418	757,106,323	829,616,791	787,877,476	873,909,559	90,924,268	911,903,341	733,170,067	862,509,969	000,108,336	0,001,083	296,537,154	0,003,283	023,439,325	117,641,187	1,293,399,024
mt-Tc	−474,097,178	−474,097,178	−481,524,977	−481,524,977	−481,524,977	−474,097,178	−474,097,178	−474,097,178	−474,097,178	−474,097,178	−478,553,857	−474,097,178	003,996,852	0,039,969	139,827,672	0,043,252	−001,349,849	099,068,719	0,044,566,793
mt-Tl1	−356,387,724	−474,097,178	−481,524,977	−481,524,977	−390,138,834	−474,097,178	−474,097,178	−474,097,178	−474,097,178	−474,097,178	−436,734,738	−474,097,178	019,609,858	0,196,099	070,752,462	017,106	011,842,552	10,855,495	−03,736,244
mt-Tn	−474,097,178	−474,097,178	−391,817,305	−481,524,977	−481,524,977	−474,097,178	−474,097,178	−474,097,178	−42,081,174	−416,948,709	−460,612,323	−452,010,397	070,539,176	0,705,392	015,156,947	0,485,758	−002,719,705	098,132,502	0,086,019,262
mt-Tp	−474,097,178	−474,097,178	−481,524,977	−481,524,977	−481,524,977	−421,415,359	−474,097,178	−474,097,178	−474,097,178	−474,097,178	−478,553,857	−463,560,814	019,843,752	0,198,438	070,237,516	017,106	−004,592,268	09,686,701	014,993,043
mt-Tq	−331,787,105	−360,669,986	−391,817,305	−481,524,977	−481,524,977	−474,097,178	−474,097,178	−474,097,178	−474,097,178	−474,097,178	−40,946,487	−474,097,178	00,699,287	0,069,929	115,534,268	0,070,628	021,144,312	115,784,579	−0,646,323,079
mt-Ts1	−15,730,264	−2,54,397,684	−336,921,733	−267,830,231	−206,110,406	−12,636,561	−203,285,447	−474,097,178	−381,985,717	−231,484,961	−244,512,539	−283,443,783	059,353,143	0,593,531	022,655,659	0,428,191	021,315,417	115,921,982	−0,389,312,436
mt-Ty	−474,097,178	−474,097,178	−481,524,977	−481,524,977	−481,524,977	−474,097,178	−474,097,178	−474,097,178	−474,097,178	−416,948,709	−478,553,857	−462,667,484	020,711,064	0,207,111	068,379,683	017,106	−004,870,559	096,680,337	015,886,373


Symbol	2405_Control_1	2405_Control_2	2405_Control_3	2405_Control_4	2405_Control_5	2405_Stiripentol_1	2405_Stiripentol_2	2405_Stiripentol_3	2405_Stiripentol_4	2405_Stiripentol_5	Mean Ctr	Mean Stiri	*t* test	*p* value	-Log *p*-value	q-value	log2FC	FC (stiri/ctr)	Difference
Ndufa1	393,127,747	364,972,048	402,941,699	418,395,147	4,135,334	361,479,507	332,289,375	365,491,953	372,466,155	375,165,252	398,594,008	361,378,448	001,573,396	0,015,734	180,316,085	0,051,413	−014,140,955	090,663,292	−03,721,556
Ndufa10	655,706,407	646,365,016	672,218,548	674,978,573	681,179,055	698,098,715	687,582,932	684,922,849	680,539,746	678,451,664	66,608,952	685,919,181	002,703,013	002,703	156,815,395	0,076,349	004,232,252	102,977,026	019,829,661
Ndufa11	545,770,145	518,472,812	564,753,172	5,504,735	564,374,506	530,362,877	52,094,415	539,660,622	542,550,182	529,105,203	548,768,827	532,524,607	011,891,074	0,118,911	092,477,797	0,200,166	−004,335,034	097,039,879	−01,624,422
Ndufa12	543,428,798	534,559,639	570,795,276	559,448,245	563,465,507	567,895,028	548,028,211	58,359,994	59,296,775	584,074,677	554,339,493	575,313,121	007,754,557	0,077,546	11,104,406	0,159,543	005,357,758	103,783,535	020,973,628
Ndufa13	678,085,363	646,948,267	675,036,757	674,260,185	677,957,647	707,745,421	703,646,866	683,530,855	679,740,136	666,505,073	670,457,644	68,823,367	01,046,452	0,104,645	098,028,152	0,190,284	003,775,228	102,651,327	017,776,026
Ndufa2	571,984,284	539,699,668	559,547,756	557,321,684	551,095,763	586,547,486	578,965,076	588,412,002	578,154,629	560,894,826	555,929,831	578,594,804	001,351,666	0,013,517	186,911,969	0,045,051	005,765,057	104,076,949	022,664,973
Ndufa3	506,847,388	492,092,655	470,512,633	474,681,908	464,929,005	52,097,272	506,898,713	487,826,063	484,088,651	481,072,172	481,812,718	496,171,664	022,363,714	0,223,637	065,045,634	0,295,787	004,236,687	102,980,192	014,358,946
Ndufa4	723,308,298	704,059,424	723,753,357	719,987,386	70,800,155	705,044,155	697,351,105	698,249,193	685,767,061	695,293,282	715,822,003	696,340,959	000,533,128	0,005,331	227,319,132	0,026,923	−0,039,807	097,278,507	−019,481,044
Ndufa4l2	−310,776,588	−193,869,836	−266,171,852	−226,564,945	−313,258,482	−38,290,913	−323,259,667	−131,952,107	−241,296,844	−196,853,182	−262,128,341	−255,254,186	089,472,422	0,894,724	004,831,091	0,866,894	−003,833,872	097,377,561	006,874,155
Ndufa5	477,985,001	454,081,995	493,050,199	482,613,475	48,169,805	552,065,558	52,512,008	568,604,155	553,508,917	557,908,962	477,885,744	551,441,534	6,2323E-05	0,000,062	420,760,831	0,001,484	02,065,422	11,539,192	07,355,579
Ndufa6	742,056,696	714,905,352	624,225,747	621,871,199	676,027,317	759,979,178	752,642,844	700,714,675	702,062,205	687,576,563	675,817,262	720,595,093	015,086,329	0,150,863	082,141,726	023,279	009,255,563	106,625,731	044,777,831
Ndufa7	730,326,138	691,157,919	691,503,144	684,547,808	701,809,995	723,175,714	717,687,853	705,841,384	703,116,523	690,974,901	699,869,001	708,159,275	042,601,708	0,426,017	037,057,307	049,997	001,698,897	101,184,547	008,290,274
Ndufa8	648,622,157	629,653,835	596,085,563	595,881,804	606,609,668	627,926,084	62,747,989	662,004,374	657,880,498	647,473,154	615,370,605	6,445,528	004,982,822	0,049,828	130,252,654	0,120,346	006,684,301	104,742,215	029,182,195
Ndufa9	650,607,129	645,852,731	646,417,788	644,086,294	657,288,013	708,272,641	698,042,462	697,278,899	691,731,867	694,060,394	648,850,391	697,877,253	9,8793E-07	0,000,001	6	0,000,165	010,508,744	107,555,958	049,026,862
Ndufab1	578,051,916	554,519,432	57,586,179	575,786,755	553,656,016	605,586,126	592,727,008	601,298,663	592,888,638	587,376,744	567,575,182	595,975,436	000,221,904	0,002,219	26,538,427	0,019,333	007,044,136	105,003,787	028,400,254
Ndufab1-ps	−356,387,724	−151,360,915	−240,614,582	−325,850,733	−278,163,806	−208,814,619	−251,144,881	−250,811,357	−381,985,717	−474,097,178	−270,475,552	−31,337,075	050,306,109	0,503,061	029,837,935	0,569,927	021,237,233	115,859,178	−042,895,198
Ndufaf1	375,097,849	377,746,002	33,026,253	327,679,035	358,869,602	250,444,662	254,842,335	344,409,155	373,012,122	354,476,951	353,931,003	315,437,045	020,885,178	0,208,852	068,016,136	0,282,969	−016,611,604	089,123,881	−038,493,959
Ndufaf2	246,575,624	214,310,837	314,171,785	309,658,078	257,024,456	20,386,136	210,932,077	262,319,323	24,015,611	260,647,548	268,348,156	235,583,284	018,691,567	0,186,916	072,835,352	0,262,975	−01,878,688	087,790,163	−032,764,872
Ndufaf3	341,269,219	313,698,838	341,873,631	357,278,808	386,539,024	389,766,268	388,431,683	388,009,388	393,627,218	375,554,089	348,131,904	387,077,729	001,316,855	0,013,169	18,804,472	0,044,787	015,298,927	111,187,089	038,945,825
Ndufaf4	21,554,608	225,539,687	379,008,545	380,231,302	328,606,017	293,587,143	280,494,342	338,320,553	31,874,361	346,627,475	305,786,326	315,554,625	080,471,496	0,804,715	00,943,579	0,798,316	004,536,587	103,194,485	009,768,299
Ndufaf5	376,105,112	363,940,946	35,319,361	343,696,863	382,440,461	443,270,187	431,270,204	404,338,161	400,993,905	39,222,139	363,875,399	41,441,877	00,030,264	0,003,026	251,913,108	002,096	018,764,483	113,890,296	050,543,371
Ndufaf6	269,319,662	209,863,979	217,712,542	217,596,293	239,614,779	412,862,919	409,117,922	29,514,574	300,730,062	28,837,509	230,821,451	341,246,347	000,681,425	0,006,814	216,659,787	0,030,214	05,640,363	147,839,963	110,424,896
Ndufaf7	473,626,526	465,526,621	448,897,674	451,514,057	471,975,407	371,235,358	401,319,034	50,165,229	498,180,865	502,238,838	462,308,057	454,925,277	080,478,349	0,804,783	009,432,121	0,798,316	−002,322,491	098,403,061	−00,738,278
Ndufaf8	331,123,515	292,413,746	350,688,933	343,006,092	355,247,243	350,597,774	334,019,637	386,818,002	381,819,958	361,493,868	334,495,906	362,949,848	009,333,905	0,093,339	102,993,686	0,175,956	011,778,166	108,506,514	028,453,942
Ndufb10	599,982,074	58,231,474	572,476,112	585,605,218	575,781,839	647,078,537	639,202,609	598,489,012	594,736,388	589,154,548	583,231,997	613,732,219	004,788,401	0,047,884	131,980,958	0,117,968	007,353,945	105,229,518	030,500,222
Ndufb11	620,497,716	600,481,292	57,744,459	576,566,205	585,377,002	679,650,103	660,178,499	596,943,958	595,826,031	583,202,694	592,073,361	623,160,257	018,032,615	0,180,326	074,394,165	0,256,849	007,382,728	105,250,514	031,086,896
Ndufb2	508,927,706	488,209,783	450,463,369	438,068,661	443,877,315	425,106,763	428,871,272	429,039,388	440,188,658	41,695,794	465,909,367	428,032,804	00,298,738	0,029,874	152,470,662	0,082,974	−012,232,797	091,870,401	−037,876,562
Ndufb3	50,785,774	501,764,479	495,635,074	503,631,561	493,940,998	498,264,393	489,341,425	496,870,724	497,262,551	489,510,024	500,565,971	494,249,823	008,761,652	0,087,617	105,741,162	0,167,831	−001,831,977	098,738,199	−006,316,147
Ndufb4	311,053,052	270,434,313	315,169,642	303,720,955	31,004,515	25,579,211	260,268,709	253,551,817	254,476,082	269,011,949	302,084,623	258,620,133	000,098,811	0,000,988	300,524,306	0,012,275	−022,411,815	085,611,817	−043,464,489
Ndufb5	621,080,701	603,953,443	625,735,427	627,697,676	6,367,372	675,549,394	660,919,568	64,077,714	635,002,025	625,852,043	62,304,089	647,620,034	004,800,023	0,048	131,875,876	0,117,968	005,582,077	103,945,029	024,579,144
Ndufb6	5,202,016	48,864,641	515,358,568	517,084,851	527,335,613	513,048,435	501,383,117	501,266,266	492,818,913	480,040,705	513,725,408	497,711,487	009,849,552	0,098,496	100,658,141	0,182,381	−004,568,774	096,882,786	−016,013,921
Ndufb7	52,318,969	500,163,329	577,928,132	566,776,049	536,908,913	505,595,736	501,306,078	559,397,113	558,415,059	543,559,773	540,993,223	533,654,752	07,097,199	070,972	014,891,296	0,742,452	−001,970,383	098,643,519	−007,338,471
Ndufb8	724,763,323	696,002,748	713,799,078	709,696,285	697,931,055	748,409,255	731,580,139	742,062,348	737,720,863	723,746,424	708,438,498	736,703,806	000,316	000,316	250,031,292	002,096	005,644,208	103,989,804	028,265,308
Ndufb9	712,007,085	690,943,538	708,690,144	709,696,285	725,392,956	744,286,708	72,959,868	765,523,814	765,789,031	753,549,044	709,346,002	751,749,455	000,130,135	0,001,301	28,857,227	0,014,458	008,376,241	105,977,824	042,403,454
Ndufc1	518,091,251	482,182,208	511,712,854	515,278,036	539,030,752	490,619,775	462,251,661	501,960,367	484,927,313	485,669,891	51,325,902	485,085,802	003,572,805	0,035,728	144,699,129	0,096,034	−008,144,712	094,510,916	−028,173,219
Ndufc2	527,938,992	506,271,206	55,391,009	551,074,951	549,998,751	486,517,664	483,445,665	502,804,202	502,168,255	49,555,069	537,838,798	494,097,295	000,233,215	0,002,332	263,227,145	0,019,333	−012,237,867	091,867,172	−043,741,503
Ndufs1	665,484,223	665,813,532	706,479,343	709,841,589	706,249,798	661,579,384	658,373,872	674,489,446	6,615,405	676,945,245	690,773,697	666,585,689	005,826,057	0,058,261	123,462,207	0,133,002	−00,514,228	096,498,418	−024,188,007
Ndufs2	834,191,546	820,259,727	764,102,069	769,176,164	779,839,472	79,441,663	781,113,774	764,492,573	754,625,959	749,608,794	793,513,795	768,851,546	017,212,674	0,172,127	0,764,151	0,249,434	−004,555,024	09,689,202	−02,466,225
Ndufs3	676,696,726	653,977,371	595,837,056	607,223,059	603,153,956	683,658,276	672,897,662	652,161,607	647,965,179	641,052,156	627,377,633	659,546,976	011,026,016	011,026	095,758,201	0,195,477	007,214,132	105,127,588	032,169,343
Ndufs4	579,202,666	566,185,675	542,339,874	546,979,664	541,600,119	648,465,942	634,688,334	578,032,715	573,070,482	575,108,191	5,552,616	601,873,133	003,210,421	0,032,104	149,344,085	0,087,708	011,629,179	108,394,518	046,611,533
Ndufs5	457,147,713	428,708,294	470,851,032	471,496,732	480,568,583	499,014,521	489,508,775	512,348,426	509,686,107	506,452,994	461,754,471	503,402,165	00,030,821	0,003,082	251,116,737	002,096	012,458,549	109,019,446	041,647,694
Ndufs6	493,025,224	465,270,523	486,983,581	493,016,179	494,162,297	499,313,484	493,305,194	502,804,202	499,698,516	492,475,662	486,491,561	497,519,412	009,397,002	009,397	102,701,077	0,175,956	003,233,804	102,266,812	011,027,851
Ndufs7	563,612,399	534,559,639	528,519,074	520,024,262	548,691,435	553,046,366	546,007,415	546,317,737	546,587,521	535,139,013	539,081,362	54,541,961	046,351,289	0,463,513	033,393,808	0,540,171	001,686,354	10,117,575	006,338,249
Ndufs8	607,509,003	586,135,669	571,722,158	564,308,207	576,408,416	685,230,876	671,364,789	609,157,558	604,952,122	583,662,289	581,216,691	630,873,527	004,813,595	0,048,136	131,753	0,117,968	011,827,468	108,543,601	049,656,836
Ndufv1	693,672,151	67,716,549	681,497,678	677,066,957	697,823,301	74,727,587	740,893,844	731,628,494	732,780,017	719,728,918	685,445,115	734,461,429	5,6998E-05	0,000,057	424,412,514	0,001,484	009,964,558	107,151,019	049,016,313
Ndufv2	686,284,154	666,704,544	673,053,163	682,148,372	66,506,915	711,224,365	69,831,808	695,781,001	687,666,665	684,621,392	674,651,877	695,522,301	001,033,295	0,010,333	198,577,357	0,040,046	004,395,352	10,309,351	020,870,424
Ndufv3	657,905,615	647,456,684	582,286,677	582,961,226	591,211,113	599,378,095	594,151,133	642,588,141	64,510,083	63,638,431	612,364,263	623,520,502	059,137,698	0,591,377	022,813,557	0,641,905	002,604,691	10,182,183	011,156,239
Sdha	785,528,239	791,830,164	763,836,181	764,305,972	784,199,228	817,954,461	814,284,542	782,211,765	775,972,187	781,661,923	777,939,957	794,416,976	016,095,723	0,160,957	079,329,013	0,246,032	003,023,764	102,118,032	016,477,019
Sdhb	635,770,633	602,969,898	608,971,336	604,045,649	609,885,423	606,085,666	606,818,328	650,275,047	642,234,678	641,236,996	612,328,588	629,330,143	016,811,089	0,168,111	077,440,387	024,771	003,951,101	102,776,541	017,001,555
Sdhc	713,563,899	708,306,022	67,689,219	669,944,907	685,293,505	691,552,373	685,469,968	672,592,264	660,702,186	656,224,542	690,800,105	673,308,267	015,011,929	0,150,119	082,356,434	023,279	−003,700,112	097,467,887	−017,491,838
Sdhd	684,854,545	664,981,207	657,144,481	647,594,155	647,390,966	704,072,187	695,718,262	627,774,862	621,571,162	624,025,193	660,393,071	654,632,333	077,856,867	0,778,569	010,870,289	0,783,427	−001,264,012	09,912,768	−005,760,738
Uqcc1	542,217,274	530,050,029	525,078,898	528,499,386	540,748,738	456,315,448	467,703,293	562,969,382	56,623,848	558,128,212	533,318,865	522,270,963	066,950,383	0,669,504	017,424,682	0,710,655	−003,019,987	097,928,462	−011,047,902
Uqcc2	551,437,671	520,059,492	614,258,252	600,951,225	599,329,246	615,629,574	606,558,494	612,700,166	599,538,468	592,559,495	577,207,177	605,397,239	016,247,759	0,162,478	078,920,544	0,246,032	006,879,286	104,883,872	028,190,062
Uqcc3	323,619,737	296,599,725	365,630,148	358,112,666	34,690,176	321,217,106	326,712,246	419,959,213	410,124,832	39,980,246	338,172,807	375,563,171	016,945,083	0,169,451	077,095,586	024,771	015,129,494	111,056,585	037,390,364
Uqcr10	614,502,466	584,350,184	652,061,395	646,781,376	649,465,634	685,313,171	670,389,456	675,255,275	665,301,688	658,071,858	629,432,211	67,086,629	001,787,144	0,017,871	174,785,115	0,056,194	009,197,424	106,582,771	041,434,079
Uqcr11	569,027,819	539,239,886	576,065,686	556,430,663	566,399,051	451,716,482	460,319,453	523,238,418	508,412,657	506,531,335	561,432,621	490,043,669	000,182,268	0,001,823	273,921,333	0,016,875	−019,620,258	087,284,502	−071,388,952
Uqcrb	627,078,578	62,891,371	602,400,913	595,039,425	60,748,602	669,756,525	659,356,327	608,937,811	60,999,435	613,373,845	612,183,729	632,283,772	021,473,337	0,214,733	066,810,121	0,288,591	004,660,749	103,283,335	020,100,042
Uqcrc1	731,982,395	719,069,409	748,018,901	741,732,512	745,023,817	703,710,531	709,933,614	734,542,765	730,922,588	725,129,974	737,165,407	720,847,894	00,752,363	0,075,236	11,235,743	0,158,711	−00,322,935	097,786,452	−016,317,512
Uqcrc2	702,226,218	690,244,592	675,671,511	684,807,166	693,260,469	774,774,069	758,799,112	722,097,284	713,787,982	71,850,873	689,241,991	737,593,435	000,593,609	0,005,936	222,650,611	0,028,264	009,781,522	107,015,162	048,351,444
Uqcrfs1	687,371,319	672,121,505	672,009,137	673,981,137	657,145,178	673,242,193	670,675,602	629,850,644	626,857,005	626,671,747	672,525,655	645,459,438	005,178,929	0,051,789	128,576,247	0,121,559	−005,926,287	095,975,437	−027,066,217
Uqcrh	71,210,488	681,295,507	633,494,145	630,682,192	631,285,311	681,351,347	667,130,581	530,740,802	531,054,977	519,481,627	657,772,407	585,951,867	010,889,402	0,108,894	096,299,605	0,195,131	−016,680,633	089,081,248	−07,182,054
Uqcrh-ps1	−331,787,105	−474,097,178	−481,524,977	−481,524,977	−481,524,977	−474,097,178	−419,379,481	−474,097,178	−474,097,178	−474,097,178	−4,50,091,843	−4,63,153,639	068,992,089	0,689,921	016,120,064	0,727,692	004,127,143	102,902,029	−013,061,796
Uqcrq	588,438,424	55,130,815	688,917,044	692,112,116	674,134,338	716,124,337	704,421,934	766,768,835	76,687,111	751,039,712	638,982,014	741,045,186	001,237,299	0,012,373	190,752,499	0,044,627	021,378,619	115,972,777	102,063,171
Cyc1	731,313,615	709,017,566	755,857,363	738,002,681	750,013,933	732,990,768	734,563,426	78,659,965	779,672,526	774,935,579	736,841,031	76,175,239	011,695,917	0,116,959	093,196,635	0,200,166	004,796,872	103,380,832	024,911,358
Cox10	41,126,578	387,296,632	480,017,619	480,937,504	50,015,405	37,986,134	384,329,773	517,482,209	517,433,565	518,114,854	451,934,317	463,444,348	07,803,714	0,780,371	010,769,888	0,783,427	0,036,283	102,546,837	011,510,031
Cox11	296,126,138	306,946,813	364,574,924	367,370,854	363,865,568	2,942,073	290,799,562	345,323,469	338,033,313	339,078,206	339,776,859	32,148,837	038,083,089	0,380,831	041,926,771	0,456,541	−007,982,105	0,946,175	−018,288,489
Cox14	576,391,901	550,742,296	544,739,469	540,375,288	549,497,338	619,218,579	62,482,027	586,797,263	584,330,762	565,807,174	552,349,258	59,619,481	000,912,159	0,009,122	203,990,993	0,037,076	011,020,302	107,938,012	043,845,551
Cox15	599,769,254	586,578,609	570,074,948	575,556,701	600,003,277	529,638,592	552,365,954	619,976,687	621,506,281	620,193,769	586,396,558	588,736,257	091,295,837	0,912,958	00,395,492	0,879,448	000,574,484	100,398,996	002,339,699
Cox16	41,540,142	386,855,894	413,126,714	402,952,219	375,227,633	433,829,033	412,151,734	363,496,939	371,184,138	368,389,457	398,712,776	38,981,026	059,355,207	0,593,552	022,654,123	0,641,905	−003,257,777	097,767,186	−008,902,516
Cox17	508,794,393	475,891,779	451,336,662	440,906,702	47,828,277	395,551,777	389,104,137	492,254,773	488,967,728	488,004,026	471,042,461	450,776,488	046,897,467	0,468,975	032,885,031	054,274	−006,344,485	095,697,633	−020,265,973
Cox18	321,437,023	32,898,438	431,008,523	420,298,717	444,502,643	473,729,031	448,704,898	445,646,458	43,730,948	430,442,345	389,246,257	447,166,442	006,793,167	0,067,932	11,679,256	0,148,958	020,012,876	114,880,088	057,920,185
Cox19	39,679,751	371,500,358	3,728,082	364,416,667	361,296,671	366,064,311	388,768,302	313,025,909	317,414,512	318,991,954	373,363,881	340,852,997	008,587,015	008,587	106,615,854	0,166,398	−01,314,327	09,129,244	−032,510,884
Cox20	297,574,992	261,244,457	329,138,614	32,482,756	266,971,355	396,772,766	382,585,615	306,316,685	298,920,026	307,947,601	295,951,396	338,508,539	013,177,377	0,131,774	088,017,027	0,215,295	019,383,197	114,379,774	042,557,143
Cox20-ps	−423,467,136	−474,097,178	−481,524,977	−42,443,283	−481,524,977	−421,415,359	−474,097,178	−474,097,178	−474,097,178	−474,097,178	−45,700,942	−463,560,814	071,282,488	0,712,825	014,701,708	0,742,452	002,053,472	101,433,536	−006,551,395
Cox4i1	841,284,268	809,163,313	822,427,323	815,937,199	825,153,361	857,067,682	846,718,079	818,505,132	818,035,861	808,761,765	822,793,093	829,817,704	053,243,825	0,532,438	027,373,096	0,591,539	001,226,475	100,853,752	007,024,611
Cox4i2	−24,828,666	−254,397,684	−336,921,733	−267,830,231	−481,524,977	−474,097,178	−37,980,278	−474,097,178	−326,217,835	−474,097,178	−317,792,257	−42,566,243	007,916,702	0,079,167	110,145,581	0,160,892	042,162,578	133,943,613	−107,870,173
Cox5a	661,050,001	647,021,008	658,554,634	653,347,006	648,178,803	699,987,486	682,603,665	677,574,231	668,686,819	665,727,765	65,363,029	678,915,993	000,532,032	000,532	227,408,837	0,026,923	005,475,823	103,868,502	025,285,703
Cox5b	710,235,351	692,808,656	704,702,688	700,117,433	704,322,027	703,039,067	695,958,372	726,933,497	727,212,404	7,187,428	702,437,231	714,377,228	012,592,967	012,593	08,998,708	0,209,862	002,431,678	101,699,796	011,939,997
Cox6a1	685,796,156	669,904,618	718,034,275	708,069,833	729,532,878	697,834,943	695,196,648	745,132,131	742,540,162	740,804,447	702,267,552	724,301,666	019,812,815	0,198,128	070,305,414	027,515	004,456,992	103,137,567	022,034,114
Cox6a2	−27,617,433	−220,982,486	−03,475,743	−06,075,916	−031,317,391	257,398,383	262,302,609	278,763,411	308,889,011	270,628,171	−124,798,159	275,596,317	6,0078E-05	000,006	422,184,875	0,001,484	#NOMBRE!	−220,833,639	400,394,476
Cox6b1	680,640,906	667,967,948	660,542,283	653,293,285	66,242,997	683,990,777	669,743,544	638,823,988	639,654,184	63,758,919	664,974,878	653,960,337	033,140,237	0,331,402	047,964,487	040,609	−00,240,967	098,343,615	−011,014,542
Cox6b2	−474,097,178	−474,097,178	−481,524,977	−481,524,977	−481,524,977	−474,097,178	−474,097,178	−474,097,178	−474,097,178	−376,123,602	−478,553,857	−454,502,463	025,642,294	0,256,423	059,104,302	0,326,205	−007,439,319	094,974,151	024,051,395
Cox6c	670,135,353	65,342,191	713,390,664	706,220,213	717,226,388	681,055,074	665,238,308	675,832,755	662,569,385	667,637,602	692,078,905	670,466,625	014,100,865	0,141,009	085,075,317	0,223,801	−004,577,101	096,877,194	−021,612,281
Cox7a1	140,443,901	059,665,224	−066,025,924	−049,813,312	−165,227,699	−173,162,986	−184,235,119	−035,616,505	−046,907,236	−069,189,286	−016,191,562	−101,822,226	020,346,853	0,203,469	069,150,175	0,277,935	265,273,846	628,859,819	−085,630,665
Cox7a2	615,142,047	59,072,007	627,200,886	610,548,938	622,018,552	575,665,305	546,571,592	537,391,587	531,902,826	531,693,891	613,126,099	54,464,504	000,016,547	0,000,165	378,251,606	0,002,757	−017,086,752	088,830,836	−068,481,058
Cox7a2l	762,051,829	735,881,153	699,133,402	696,718,104	740,015,906	80,368,999	786,496,506	737,774,968	737,764,354	732,690,822	726,760,079	759,683,328	012,784,594	0,127,846	089,331,286	0,210,946	006,391,899	10,453,014	032,923,249
Cox7b	659,931,515	647,818,745	638,152,905	642,415,776	627,678,936	666,512,955	646,256,823	598,133,927	592,690,669	590,517,434	643,199,575	618,822,362	018,002,004	018,002	074,467,924	0,256,849	−005,574,112	096,210,008	−024,377,214
Cox7c	626,105,947	597,231,926	621,723,468	616,771,676	62,012,236	652,188,036	638,549,289	581,753,215	57,491,969	579,330,466	616,391,075	605,348,139	054,024,247	0,540,242	026,741,166	0,596,234	−002,608,089	098,208,453	−011,042,936
Cox8a	799,198,985	772,644,972	756,570,481	750,908,716	769,418,725	853,169,257	841,411,472	812,239,812	809,979,286	799,660,214	769,748,376	823,292,008	000,364,734	0,003,647	243,806,424	002,096	00,970,173	106,955,992	053,543,633
Atp10a	466,116,154	479,420,265	504,802,197	506,335,201	462,011,169	423,474,126	442,121,124	477,083,371	475,154,248	485,125,839	483,736,997	460,591,741	016,387,362	0,163,874	078,548,995	0,246,032	−007,073,434	095,215,322	−023,145,256
Atp10b	−474,097,178	−297,962,049	−391,817,305	−42,443,283	−237,684,836	−38,290,913	−474,097,178	−348,532,528	−381,985,717	−318,309,201	−36,519,884	−381,166,751	075,882,168	0,758,822	011,986,009	0,771,083	00,617,401	104,372,388	−015,967,911
Atp10 d	364,799,464	378,215,421	408,645,216	414,370,283	443,877,315	201,527,502	228,808,165	322,137,875	306,316,469	335,534,923	40,198,154	278,864,987	000,354,154	0,003,542	245,075,144	002,096	−052,756,244	069,372,585	−123,116,553
Atp11a	790,097,779	805,136,817	788,942,023	781,099,123	80,769,768	586,098,146	619,077,001	771,525,472	777,498,952	788,021,816	794,594,685	708,444,277	008,536,583	0,085,366	106,871,507	0,166,398	−016,556,476	089,157,943	−086,150,407
Atp11b	691,771,236	703,814,346	677,135,091	681,133,016	699,591,034	692,768,476	699,298,153	654,131,036	660,677,456	681,418,117	690,688,945	677,658,648	023,720,848	0,237,208	062,487,067	0,308,834	−002,747,739	098,113,435	−013,030,297
Atp11c	446,766,828	461,373,475	50,319,047	509,935,587	50,752,843	388,805,822	390,106,979	444,962,803	445,560,619	45,025,232	485,758,958	423,937,708	001,258,621	0,012,586	190,011,227	0,044,627	−01,963,883	087,273,266	−06,182,125
Atp13a1	612,404,024	59,835,717	67,820,304	675,831,346	648,153,456	625,860,108	638,638,553	653,621,027	64,944,316	659,217,931	642,589,807	645,356,156	087,680,314	0,876,803	005,709,797	0,859,525	000,619,747	1,004,305	002,766,349
Atp13a2	524,451,594	507,997,083	468,722,898	483,662,039	477,870,751	366,627,301	380,642,367	485,775	480,425,793	488,803,277	492,540,873	440,454,748	011,304,534	0,113,045	094,674,864	019,624	−016,124,965	089,425,015	−052,086,126
Atp13a3	774,394,058	782,783,446	770,700,587	76,590,258	824,878,091	587,036,085	602,718,277	755,648,187	747,718,658	760,286,766	783,731,753	690,681,594	005,139,327	0,051,393	128,909,603	0,121,559	−018,233,917	088,127,295	−093,050,158
Atp13a4	−292,440,541	−474,097,178	−391,817,305	−481,524,977	−390,138,834	−35,254,519	−282,742,491	−0,489,962	−057,281,307	−01,362,777	−406,003,767	−151,038,592	001,093,426	0,010,934	196,122,093	0,040,669	−142,657,589	037,201,278	254,965,175
Atp1a1	958,235,432	964,419,421	939,106,246	936,613,893	927,774,452	999,139,939	10,034,544	965,485,165	96,260,701	963,504,345	945,229,889	978,838,172	001,928,072	0,019,281	171,487,045	0,058,421	005,040,511	103,555,567	033,608,283
Atp1a2	−27,617,433	−171,055,353	−191,468,916	−115,403,269	−107,355,176	−421,415,359	−37,980,278	−114,380,655	−156,429,808	−15,295,101	−172,291,409	−244,995,922	033,684,784	0,336,848	047,256,603	040,975	050,790,697	142,198,572	−072,704,514
Atp1a3	−055,221,267	−104,600,519	−131,095,176	−076,778,939	−33,463,208	−082,411,862	−213,845,322	−032,456,854	−050,283,048	−045,779,233	−140,465,596	−084,955,264	038,353,271	0,383,533	041,619,726	0,456,541	−072,544,158	06,048,119	055,510,332
Atp1a4	−386,063,403	−474,097,178	−297,252,592	−325,850,733	−313,258,482	−474,097,178	−474,097,178	−348,532,528	−474,097,178	−376,123,602	−359,304,478	−429,389,533	013,883,105	0,138,831	085,751,355	0,222,463	025,708,011	119,505,756	−070,085,055
Atp1b1	819,478,478	816,130,379	723,342,847	726,747,207	785,114,462	743,340,609	777,875,697	765,907,093	753,538,616	765,258,614	774,162,675	761,184,126	056,717,125	0,567,171	024,628,598	0,621,836	−002,439,128	098,323,537	−012,978,549
Atp1b2	−022,615,762	015,053,788	−040,492,334	−046,341,778	012,099,865	−230,601,491	−203,285,447	−004,167,877	−001,485,097	000,107,544	−016,459,244	−087,886,474	022,570,438	0,225,704	064,646,074	029,617	241,674,307	533,964,218	−07,142,723
Atp1b3	577,886,772	582,086,796	721,599,891	728,129,162	69,148,804	493,524,467	498,190,268	615,073,037	600,667,567	608,435,303	660,238,132	563,178,128	0,055,198	0,055,198	125,807,666	012,776	−022,939,516	085,299,243	−097,060,004
Atp23	223,470,106	179,851,233	260,877,502	231,243,518	193,337,018	157,904,207	175,851,346	221,376,416	216,943,256	19,259,663	217,755,875	192,934,371	022,155,052	0,221,551	065,452,629	0,295,371	−017,460,146	088,601,224	−024,821,504
Atp2a1	−150,707,421	−193,869,836	−252,828,023	−267,830,231	−278,163,806	−181,284,358	−213,845,322	−250,811,357	−179,577	−196,853,182	−228,679,863	−204,474,244	040,709,299	0,407,093	039,030,637	0,481,149	−016,141,021	089,415,063	02,420,562
Atp2a2	846,364,687	848,656,857	897,333,867	896,682,412	910,545,578	706,098,687	745,021,815	852,653,206	852,375,978	861,133,718	87,991,668	803,456,681	006,109,738	0,061,097	121,398,011	0,135,758	−013,114,668	091,310,541	−0,7646
Atp2a3	028,273,745	−069,343,267	204,951,434	23,224,172	296,776,304	355,939,654	374,464,944	605,413,132	616,487,979	619,085,252	138,579,987	514,278,192	000,343,917	0,003,439	246,356,782	002,096	189,183,005	371,105,671	375,698,205
Atp2b1	597,551,539	617,865,556	729,310,702	725,335,582	721,771,287	498,639,944	538,405,404	649,668,311	643,688,784	659,054,763	678,366,933	597,891,441	010,504,755	0,105,048	097,861,221	0,190,284	−018,218,229	088,136,879	−080,475,492
Atp2b2	−474,097,178	−474,097,178	−481,524,977	−481,524,977	−481,524,977	−474,097,178	−474,097,178	−37,959,928	−474,097,178	−474,097,178	−478,553,857	−455,197,598	025,359,523	0,253,595	059,585,931	032,509	−0,07,218,835	095,119,409	023,356,259
Atp2b3	−474,097,178	−474,097,178	−481,524,977	−42,443,283	−481,524,977	−38,290,913	−419,379,481	−474,097,178	−381,985,717	−474,097,178	−467,135,428	−426,493,737	011,839,344	0,118,393	092,667,397	0,200,166	−013,131,631	091,299,805	040,641,691
Atp2b4	174,614,559	244,493,821	−062,575,938	−021,220,752	−098,122,391	−091,087,418	−045,935,184	−001,621,659	−028,160,078	007,761,893	04,743,786	−031,808,489	029,314,891	0,293,149	053,291,158	03,701	#NOMBRE!	−06,705,296	−079,246,349
Atp2c1	608,381,053	621,382,704	611,734,515	610,187,184	606,169,487	479,838,366	483,271,128	578,576,588	57,907,195	588,756,448	611,570,989	541,902,896	00,230,656	0,023,066	163,702,771	0,068,641	−017,448,561	088,608,339	−069,668,093
Atp2c2	−002,814,569	−032,708,539	−218,910,691	−239,052,959	−215,887,023	−100,318,242	−084,987,265	017,275,856	025,127,232	066,210,596	−141,874,756	−015,338,365	007,036,581	0,070,366	115,263,714	0,152,292	−320,940,133	010,811,201	126,536,392
Atp4a	−474,097,178	−474,097,178	−429,808,059	−481,524,977	−481,524,977	−38,290,913	−474,097,178	−419,245,583	−474,097,178	−474,097,178	−468,210,474	−444,889,249	030,250,553	0,302,506	051,926,601	0,376,213	−007,371,097	095,019,072	023,321,224
Atp5a1	965,321,923	960,325,024	976,007,628	960,388,359	970,188,351	981,243,272	978,462,292	959,243,875	950,917,178	950,375,984	966,446,257	96,404,852	07,511,023	0,751,102	012,430,108	0,767,921	−000,358,375	099,751,902	−002,397,737
Atp5b	998,736,893	990,749,937	101,269,244	100,653,535	100,293,318	104,852,396	1,035,461	101,892,794	101,052,147	100,694,735	100,232,956	102,407,634	003,646,849	0,036,468	143,808,805	0,096,468	003,096,633	102,169,624	021,746,784
Atp5c1	841,563,507	824,741,687	78,079,625	783,831,507	792,215,856	873,505,798	853,241,075	813,993,392	807,014,297	803,580,008	804,629,761	830,266,914	020,301,949	0,203,019	069,246,332	0,277,935	004,525,011	103,186,205	025,637,152
Atp5c1-ps	−261,557,697	−474,097,178	−481,524,977	−481,524,977	−42,871,378	−35,254,519	−419,379,481	−322,984,704	−42,081,174	−474,097,178	−425,483,722	−397,963,659	059,703,117	0,597,031	022,400,312	0,641,905	−0,09,646,724	093,532,053	027,520,063
Atp5d	73,548,855	719,817,757	738,135,422	726,666,452	730,681,954	719,863,174	712,879,396	697,159,638	698,414,967	683,431,356	730,158,027	702,349,706	000,469,995	00,047	232,790,214	0,025,266	−00,560,192	096,191,465	−027,808,321
Atp5e	515,631,922	474,696,175	526,751,895	519,278,001	522,632,883	53,889,556	52,174,894	501,806,411	494,315,385	492,906,689	511,798,175	509,934,597	088,933,758	0,889,338	005,093,315	0,866,714	−000,526,278	099,635,876	−001,863,578
Atp5f1	84,148,378	832,345,454	836,674,937	837,261,066	840,816,529	867,215,298	853,308,264	849,761,657	846,262,186	847,265,344	837,716,353	85,276,255	000,673,568	0,006,736	217,159,792	0,030,214	002,568,224	101,796,097	015,046,197
Atp5g1	615,683,471	599,574,781	681,242,362	677,476,487	644,897,632	662,087,651	660,255,338	689,942,785	681,698,847	67,857,055	643,774,947	674,511,034	01,125,437	0,112,544	094,867,765	019,624	006,728,561	104,774,353	030,736,087
Atp5g2	804,310,314	796,058,751	776,858,562	778,922,577	773,558,779	874,290,515	865,544,075	789,007,793	789,062,555	77,222,923	785,941,797	818,026,834	018,778,267	0,187,783	072,634,373	0,262,975	005,772,569	104,082,368	032,085,037
Atp5g3	787,792,537	772,949,092	794,660,098	794,068,826	77,379,241	749,612,856	744,122,596	725,437,479	720,998,587	720,779,313	784,652,592	732,190,166	000,014,193	0,000,142	384,771,166	0,002,628	−0,09,983,564	093,313,929	−0,52,462,426
Atp5h	733,240,376	713,902,475	70,244,641	717,512,738	684,132,694	755,750,019	741,230,216	738,597,942	737,299,776	731,789,704	710,246,939	740,933,531	000,980,362	0,009,804	20,085,967	0,038,899	006,102,342	104,320,553	030,686,593
Atp5j	643,785,606	626,250,971	655,978,249	658,075,793	641,752,247	598,517,289	590,127,612	60,359,895	594,306,053	593,591,206	645,168,573	596,028,222	4,5254E-05	0,000,045	434,678,749	0,001,484	−0,11,429,552	092,383,332	−0,49,140,351
Atp5j2	639,699,264	616,434,332	656,842,166	653,936,621	637,448,797	632,001,109	630,249,083	595,422,436	58,806,075	574,571,611	640,872,236	604,060,998	00,268,481	0,026,848	157,108,806	0,076,349	−008,534,253	094,256,072	−036,811,238
Atp5k	469,132,267	44,074,203	515,668,582	511,020,021	490,050,205	472,025,149	459,703,867	444,390,604	432,579,832	430,972,233	485,322,621	447,934,337	004,745,294	0,047,453	132,373,633	0,117,968	−011,565,684	0,922,962	−037,388,284
Atp5k-ps2	−474,097,178	−474,097,178	−481,524,977	−481,524,977	−42,871,378	−474,097,178	−474,097,178	−474,097,178	−351,423,729	−474,097,178	−467,991,618	−449,562,488	050,614,595	0,506,146	029,572,419	0,569,927	−005,796,103	096,062,081	01,842,913
Atp5l	620,189,933	596,304,699	64,671,776	62,938,802	623,650,009	643,934,618	637,533,759	618,543,589	599,991,168	594,997,869	623,250,084	619,000,201	074,654,416	0,746,544	012,694,459	0,767,921	−00,098,713	099,318,109	−004,249,884
Atp5l2-ps	−331,787,105	−474,097,178	−391,817,305	−325,850,733	−359,732,278	−421,415,359	−37,980,278	−322,984,704	−381,985,717	−416,948,709	−37,665,692	−384,627,454	081,116,112	0,811,161	009,089,294	0,799,882	003,021,071	102,116,126	−007,970,534
Atp5l-ps1	−474,097,178	−474,097,178	−481,524,977	−481,524,977	−481,524,977	−38,290,913	−419,379,481	−419,245,583	−42,081,174	−474,097,178	−478,553,857	−4,23,288,622	000,552,792	0,005,528	225,743,197	0,027,095	−017,703,959	088,451,617	055,265,235
Atp5o	695,383,776	679,854,723	686,226,781	684,979,813	679,352,902	713,796,487	701,488,894	694,328,238	688,932,869	680,060,179	685,159,599	695,721,333	01,378,359	0,137,836	086,063,734	0,222,463	002,206,948	1,015,415	010,561,734
Atp5s	282,706,787	303,054,486	264,807,632	269,702,887	297,354,236	280,588,971	2,787,029	25,181,967	250,284,808	257,601,548	283,525,206	263,799,579	008,293,852	0,082,939	108,124,121	0,164,544	−010,403,473	093,042,726	−019,725,626
Atp6ap1	762,626,958	755,533,831	713,752,012	716,476,198	751,891,018	821,534,801	819,217,412	801,235,992	804,225,035	805,909,295	740,056,003	810,424,507	000,022,832	0,000,228	364,206,515	0,003,459	013,104,335	109,508,538	070,368,503
Atp6ap2	678,764,436	670,832,464	616,245,929	627,986,116	664,051,054	690,900,888	69,311,152	719,276,217	71,088,356	715,614,709	651,576	705,957,379	000,413,186	0,004,132	238,383,969	0,022,952	011,564,762	10,834,613	054,381,379
Atp6v0a1	764,746,532	759,922,506	687,003,259	699,981,432	766,323,231	793,651,852	793,303,392	794,943,798	795,722,145	799,073,775	735,595,392	795,338,992	000,883,904	0,008,839	205,359,687	0,036,826	011,265,746	108,121,802	0,597,436
Atp6v0a2	491,454,525	472,518,798	577,040,396	576,428,961	552,971,174	457,017,848	467,605,989	572,100,129	572,068,366	58,329,473	534,082,771	530,417,412	092,030,415	0,920,304	003,606,869	0,881,429	−000,993,521	09,931,371	−003,665,358
Atp6v0a4	319,468,714	305,402,461	183,715,557	188,408,081	170,925,906	175,138,024	13,141,751	27,984,365	241,969,486	26,572,415	233,584,144	218,818,564	074,020,837	0,740,208	013,064,623	0,766,185	−0,09,420,721	093,678,689	−01,476,558
Atp6v0b	71,320,861	666,894,762	56,000,397	565,596,752	631,171,293	755,838,352	760,917,425	725,372,081	726,618,588	720,495,215	627,375,077	737,848,332	000,688,947	0,006,889	216,184,382	0,030,214	023,399,608	117,608,805	110,473,255
Atp6v0c	799,750,298	782,527,817	815,434,305	809,940,858	818,490,869	769,604,451	769,549,587	806,958,955	812,875,953	803,370,017	805,228,829	792,471,792	029,917,883	0,299,179	052,406,889	0,374,873	−0,02,303,924	098,415,725	−012,757,037
Atp6v0c-ps1	−386,063,403	−474,097,178	−361,774,101	−351,869,476	−481,524,977	−421,415,359	−474,097,178	−474,097,178	−351,423,729	−416,948,709	−411,065,827	−427,596,431	065,743,435	0,657,434	018,214,784	0,702,317	005,688,037	104,021,401	−016,530,604
Atp6v0d1	709,324,647	683,288,733	600,864,961	611,197,817	65,539,593	758,091,383	754,386,844	735,521,576	730,053,651	72,736,727	652,014,418	741,084,145	000,336,018	000,336	247,366,072	002,096	018,473,349	113,660,699	089,069,727
Atp6v0d2	−204,826,778	−474,097,178	−481,524,977	−481,524,977	−481,524,977	−421,415,359	−301,583,355	−474,097,178	−474,097,178	−474,097,178	−424,699,777	−42,905,805	094,767,938	0,947,679	002,333,874	0,902,462	00,147,295	101,026,201	−004,358,272
Atp6v0e	61,647,601	594,641,436	604,691,317	600,099,174	608,390,479	598,141,417	602,259,692	610,903,584	600,179,375	612,361,964	604,859,683	604,769,207	098,510,072	0,985,101	000,651,924	0,932,767	−000,021,582	099,985,042	−000,090,477
Atp6v0e2	−27,617,433	−297,962,049	−148,554,763	−109,970,763	−073,514,915	−327,474,005	−213,845,322	−167,036,149	−228,998,797	−160,727,126	−181,235,364	−21,961,628	049,763,004	049,763	030,309,345	0,568,014	027,712,051	121,177,388	−038,380,916
Atp6v1a	761,670,984	758,283,951	706,165,455	707,628,082	748,036,013	831,181,742	832,155,557	795,528,205	790,155,416	79,655,709	736,356,897	809,115,602	000,146,588	0,001,466	283,386,603	0,014,549	013,594,066	109,880,902	072,758,705
Atp6v1b1	−474,097,178	−474,097,178	−429,808,059	−42,443,283	−33,463,208	−474,097,178	−474,097,178	−474,097,178	−474,097,178	−416,948,709	−427,413,465	−462,667,484	024,231,958	024,232	061,561,074	0,313,043	011,434,335	108,248,224	−035,254,019
Atp6v1b2	757,032,592	741,580,538	671,988,179	69,006,543	707,397,578	768,684,161	782,360,369	793,625,255	793,552,673	78,825,536	713,612,863	785,295,563	000,243,616	0,002,436	261,332,272	0,019,333	013,809,413	11,004,504	0,716,827
Atp6v1c1	734,180,212	714,313,585	674,564	681,531,181	692,311,662	726,630,306	723,568,163	751,205,528	745,317,802	747,310,051	699,380,128	73,880,637	001,292,724	0,012,927	188,850,225	0,044,787	00,791,195	105,637,312	039,426,242
Atp6v1c2	−474,097,178	−360,669,986	−481,524,977	−42,443,283	−481,524,977	−38,290,913	−419,379,481	−419,245,583	−42,081,174	−376,123,602	−44,444,999	−403,693,907	014,886,526	0,148,865	08,272,074	023,279	−013,875,928	090,829,996	040,756,082
Atp6v1d	652,983,057	62,891,371	556,219,719	561,949,301	605,319,193	64,933,954	644,125,004	662,232,542	6,654,214	666,556,744	601,076,996	657,535,046	001,909,613	0,019,096	171,905,759	0,058,421	012,951,798	109,392,815	05,645,805
Atp6v1e1	688,411,954	673,762,097	595,160,372	595,761,765	636,874,319	693,080,652	685,276,337	672,379,917	67,347,305	667,663,238	637,994,101	678,374,639	007,633,292	0,076,333	111,728,767	0,159,011	008,853,915	106,329,296	040,380,538
Atp6v1f	708,106,948	683,458,307	573,392,292	560,882,724	603,015,377	74,145,747	731,220,469	711,765,828	713,136,128	694,039,045	62,577,113	718,323,788	001,687,628	0,016,876	177,273,048	0,054,085	019,899,919	114,790,177	092,552,659
Atp6v1g1	724,837,929	699,532,501	662,121,895	677,362,845	686,542,217	800,763,209	797,873,207	781,382,288	781,493,055	77,069,371	690,079,477	786,441,094	4,2887E-05	0,000,043	436,653,154	0,001,484	018,857,618	113,963,843	096,361,617
Atp6v1g2	106,825,759	146,652,543	172,132,761	199,433,318	107,591,137	−021,506,526	−068,078,579	07,929,927	034,913,532	094,691,243	146,527,104	023,863,788	000,868,428	0,008,684	206,128,019	0,036,826	−261,827,258	016,286,262	−122,663,316
Atp6v1h	659,509,837	635,678,198	613,600,033	62,093,287	630,771,517	71,487,297	708,125,809	697,159,638	688,892,197	697,394,518	632,098,491	701,289,026	6,2016E-05	0,000,062	420,760,831	0,001,484	014,985,978	110,946,164	069,190,535
Atp7a	552,722,104	572,050,403	483,620,003	481,468,864	513,709,737	465,379,305	493,060,624	486,633,156	475,154,248	489,774,164	520,714,222	482,000,299	007,483,668	0,074,837	112,588,363	0,158,711	−0,11,145,777	092,565,227	−0,38,713,923
Atp7b	171,193,723	170,272,743	064,792,908	−007,486,081	05,656,096	−074,228,544	−104,144,083	−012,087,801	040,481,738	042,878,291	091,066,851	−02,142,008	003,959,794	0,039,598	140,232,675	0,103,109	#NOMBRE!	−02,352,127	−112,486,931
Atp8a1	55,457,902	572,538,692	447,811,316	460,579,592	484,719,908	592,695,581	594,232,088	491,181,004	478,864,456	497,231,136	504,045,706	530,840,853	047,796,778	0,477,968	032,060,118	0,549,333	007,472,484	105,316,015	026,795,147
Atp8a2	−423,467,136	−3,60,669,986	−3,1,572,798	−4,2,443,283	−481,524,977	−474,097,178	−4,74,097,178	−4,19,245,583	−42,081,174	−3,44,339,515	−4,01,164,582	−426,518,239	051,568,879	0,515,689	028,761,213	0,576,775	00,884,132	106,320,014	−025,353,657
Atp8b1	−474,097,178	−4,74,097,178	−4,29,808,059	−4,2,443,283	−42,871,378	−38,290,913	−474,097,178	−37,959,928	−381,985,717	−474,097,178	−446,229,805	−418,537,697	030,729,965	03,073	051,243,744	0,379,344	−0,09,242,931	093,794,205	027,692,108
Atp8b2	694,318,696	691,425,447	722,592,118	722,586,587	681,078,206	570,014,462	613,379,357	618,509,294	621,213,957	629,471,183	702,400,211	610,517,651	000,013,655	0,000,137	386,327,943	0,002,628	−0,20,226,027	086,918,774	−09,188,256
Atp8b3	149,573,797	134,488,503	−266,171,852	−3,25,850,733	−278,163,806	158,697,906	153,632,713	029,983,513	0,827,852	075,825,991	−1,17,224,818	100,185,065	008,153,061	0,081,531	108,867,723	01,637	#NOMBRE!	−085,464,039	217,409,883
Atp8b4	−116,439,152	−151,360,915	14,411,997	110,868,529	066,895,404	−421,415,359	−37,980,278	−202,926,434	−351,423,729	−207,499,401	010,816,767	−312,613,541	000,269,771	0,002,698	256,895,805	0,020,435	#NOMBRE!	−289,008,293	−3,23,430,308
Atp8b5	−030,802,894	−080,165,768	−12,052,877	−25,272,526	−122,416,646	−13,826,161	−099,111,175	−071,725,923	−025,259,402	−069,189,286	−121,327,867	−080,709,479	035,404,423	0,354,044	045,094,276	0,427,547	−058,810,092	066,521,798	040,618,388
Atp9a	558,553,666	551,449,268	532,707,049	539,195,191	536,029,306	462,709,811	471,826,468	528,645,912	523,063,062	523,784,167	543,586,896	502,005,884	002,494,785	0,024,948	160,296,426	007,294	−01,148,064	092,350,623	−0,41,581,012
Atp9b	584,861,997	595,371,474	619,116,561	625,336,558	623,108,238	555,798,061	570,488,912	552,301,751	55,926,673	58,064,841	609,558,966	563,700,773	000,148,415	0,001,484	28,285,661	0,014,549	−011,283,624	092,476,824	−045,858,193
Atpaf1	447,074,028	429,367,306	409,810,964	415,352,132	396,593,451	357,344,688	369,555,855	413,713,776	410,965,045	425,720,875	419,639,576	395,460,048	016,868,185	0,168,682	077,293,126	024,771	−0,08,561,879	094,238,025	−024,179,528
Atpaf2	43,646,415	416,659,823	482,142,949	490,547,244	454,738,372	485,535,271	487,487,651	546,995,017	547,077,542	544,586,153	456,110,508	522,336,327	001,098,167	0,010,982	195,931,856	0,040,669	019,559,563	114,519,687	066,225,819
Atpif1	650,032,039	63,257,694	610,744,575	605,212,592	581,365,686	606,121,282	587,777,052	585,337,017	581,618,401	58,064,841	615,986,366	588,300,432	006,023,939	0,060,239	122,012,225	0,135,661	−006,634,532	095,505,431	−027,685,934

Mitochondrial and nuclear genes were listed, as presented in Figure 3.

**Table 3:** Ligand-contact residues in human Complex I across four conditions as predicted by CB-Dock2

Prediction	Module	Chain	Residues in contact (CurPockets)
Stiri., A		NDUFA6	LYS31, VAL34, LEU37, TRP41, VAL45, THR48, ILE56, GLY61, LYS64, VAL65, MET68, PHE69, MET70, LYS71, ASN72, ALA73, VAL75, VAL81, LEU84, VAL85, LYS87, GLY88, GLU91, THR95, LYS100, VAL105, PHE108, PHE109, GLU113, THR114
		[W]	
Stiri., A	Q	NDUFS3	GLU158, ARG175, GLU186, PRO187, GLU189
		[C]	
Stiri., A	Q-assoc	ND-ACP	LEU45
		[T]	
Stiri., D	N	NDUFS4	ASNSO, ASN51, THR52, LYS54, LYS56, THR85
Stiri., D	N	NDUFS6	VAL62, VAL63, PRO64, PRO65, ILE67, LYS73
		[RI	
Stiri., D	Q	NDUFAS	TRP111, LYS112, TRP113, PRO114, ILE115
		[V]	
Stiri., D	Q	NDUFS2	GLU92, LEU125, PRO359, PRO360, GLY361, ALA362, TYR377, VAL379, ASP381, GLY382, SER383, ARG385, PRO386, TYR387
		[D]	
Stiri., D	Q	NDUFS3	GLN39, GLN41, SER43, CYS44, LEU45, GLU49, CYS51, GLU91, ARG106, LYS108, ASN200, SER201, GLU204, PHE206, TYR209, ARG210, GLN211
		[C]	
Rote., A	P	ND1 [H]	ARG62, MET68
Rote., A	P	ND3 [A]	PHE22, TRP23, LEU24, PRO25, GLN26, MET27, ASN28, LEU29
Rote., A	Q	NDUFAS	THR27, ASP52, ASP55, ARG97, GLU98, TRP99, GLU175, ASP176, ARG177, ASN180, HIS181, ASN184, TYR185, TRP187, PHE188, SER273, PHE275, GLU276, PRO277, TRP278, THR279, LYS283, ARG320
		[P]	
Rote., A	Q	NDUFS7	LYS182, LEU183, ILE185, TRP186, ARG189
		[B]	
Rote., D	P	ND1 [H]	THR21, GLU24, ARG25, LEU28, GLN32, ARG34, SER115, ILE116, SER119, TYR127, SER128, GLY131, ALA132, ASN194, ARG195, ALA 196, ASP 199, LEU200, THR201, GLU202, GLY203, ALA216, PHE220, PHE223, PHE224, GLU227, TYR228, ARG274, ARG279
Rote., D	Q	NDUFS2	PHES1, VAL61, LEU62, LEU159, ASP160, GLY 162, MET164, PHE168, LEU411, ALA412, VAL415, ILE418
		[D]	
Rote., D	Q	NDUFS7	TRP56, PRO57, MET58, THR59, PHE60, GLY61, LEU62, MET70, ASP80, VAL85, PHE86, ARG87, ALA88, SER89, PRO90, GLN92

Pockets were defined with CurPocket on the solvent-accessible surface; contact residues were then parsed to list interacting residues (residue name + position) by subunit (short gene name with pdb chain in brackets) and functional module. Predictions: Stiripentol/Active (PDB 6G2J), Stiripentol/Deactive (6G72), Rotenone/Active (6G2J), Rotenone/Deactive (6G72). Modules include N-, Q-, and P-modules; ND-ACP is noted as Q-module-associated. This summary highlights subunit-specific interaction patterns that differ between active/deactive states and between stiripentol and rotenone.

## Data availability

All data generated or analyzed during this study are included in this published article and its supporting information files. Raw data is available upon request. Bulk RNA sequencing data are available in GEO under GSE317932.

## Supporting information

This article contains supporting information.

## Conflict of interest

The authors declare that they do not have any conflicts of interest with the content of this article.

## References

[1] Brandt U. (2006). Energy converting NADH:quinone oxidoreductase (complex I). Annu. Rev. Biochem..

[2] Hirst J. (2013). Mitochondrial complex i. Annu. Rev. Biochem..

[3] Sazanov L.A. (2015). A giant molecular proton pump: Structure and mechanism of respiratory complex I. Nat. Rev. Mol. Cell Biol..

[4] Bridges H.R., Fedor J.G., Blaza J.N., Di Luca A., Jussupow A., Jarman O.D. (2020). Structure of inhibitor-bound mammalian complex I. Nat. Commun..

[5] Kampjut D., Sazanov L.A. (2020). The coupling mechanism of mammalian respiratory complex I. Science.

[6] Degli Esposti M. (1998). Inhibitors of NADH-ubiquinone reductase: an overview. Biochim. Biophys. Acta.

[7] Majander A., Finel M., Wikström M. (1994). Diphenyleneiodonium Inhibits Reduction of Iron-Sulfur Clusters in the Mitochondrial NADH-Ubiquinone Oxidoreductase (Complex I). J. Biol. Chem..

[8] Kotlyar A.B., Karliner J.S., Cecchini G. (2005). A novel strong competitive inhibitor of complex I. FEBS Lett..

[9] Chiron C. (2016). Stiripentol and vigabatrin current roles in the treatment of epilepsy. Expert Opin. Pharmacother..

[10] Wheless J., Weatherspoon S. (2025). Use of Stiripentol in Dravet Syndrome: A Guide for Clinicians. Pediatr. Neurol..

[11] García-Peñas J.J., Calvo-Medina R., García-Ron A., Gil-Nagel A., Villanueva V., Sánchez-Carpintero R. (2025). Use of Stiripentol in Patients with Dravet Syndrome: Common Practice Among Experts in Spain. Neurol. Ther..

[12] Poisson M., Huguet F., Savattier A., Bakri-Logeais F., Narcisse G. (1984). A new type of anticonvulsant, stiripentol. Pharmacological profile and neurochemical study. Arzneimittelforschung.

[13] Quilichini P.P., Chiron C., Ben-Ari Y., Gozlan H. (2006). Stiripentol, a putative antiepileptic drug, enhances the duration of opening of GABAA-receptor channels. Epilepsia.

[14] Bacq A., Depaulis A., Castagné V., Le Guern M.E., Wirrell E.C., Verleye M. (2024). An Update on Stiripentol Mechanisms of Action: A Narrative Review. Adv. Ther..

[15] Sada N., Lee S., Otsuki T., Inoue T. (2015). Targeting LDH enzymes with a stiripentol analog to treat epilepsy. Science (1979).

[16] Guyon J., Fernandez-Moncada I., Larrieu C.M., Bouchez C.L., Pagano Zottola A.C., Galvis J. (2022). Lactate dehydrogenases promote glioblastoma growth and invasion via a metabolic symbiosis. EMBO Mol. Med..

[17] Yue Q., Wang Z., Shen Y., Lan Y., Zhong X., Luo X. (2024). Histone H3K9 Lactylation Confers Temozolomide Resistance in Glioblastoma via LUC7L2-Mediated MLH1 Intron Retention. Adv. Sci..

[18] Rico-Molina M., Ortega-Vidal J., Molina-Canteras J., Cobo J., Altarejos J., Salido S. (2024). Synthesis and hLDHA Inhibitory Activity of New Stiripentol-Related Compounds of Potential Use in Primary Hyperoxaluria. Int. J. Mol. Sci..

[19] Bongard R.D., Yan K., Hoffmann R.G., Audi S.H., Zhang X., Lindemer B.J. (2013). Depleted energy charge and increased pulmonary endothelial permeability induced by mitochondrial complex i inhibition are mitigated by coenzyme Q 1 in the isolated perfused rat lung. Free Radic. Biol. Med..

[20] Yang M., Dart C., Kamishima T., Quayle J.M. (2020). Hypoxia and metabolic inhibitors alter the intracellular ATP:ADP ratio and membrane potential in human coronary artery smooth muscle cells. PeerJ.

[21] Heldt H.W., Klingenberg M., Milovancev M. (1972). Differences between the ATP/ADP Ratios in the Mitochondrial Matrix and in the Extramitochondrial Space. Eur. J. Biochem..

[22] Lobo-Jarne T., Nývltová E., Pérez-Pérez R., Timón-Gómez A., Molinié T., Choi A. (2018). Human COX7A2L Regulates Complex III Biogenesis and Promotes Supercomplex Organization Remodeling without Affecting Mitochondrial Bioenergetics. ell Rep.

[23] Molinié T., Cougouilles E., David C., Cahoreau E., Portais J.C., Mourier A. (2022). MDH2 produced OAA is a metabolic switch rewiring the fuelling of respiratory chain and TCA cycle. Biochim. Biophys. Acta Bioenerg..

[24] Velázquez I., Pardo J.P. (2001). Kinetic characterization of the rotenone-insensitive internal NADH: Ubiquinone oxidoreductase of mitochondria from Saccharomyces cerevisiae. Arch. Biochem. Biophys..

[25] Avéret N., Jobin M.L., Devin A., Rigoulet M. (2015). Proton pumping complex I increases growth yield in Candida utilis. Biochim. Biophys. Acta Bioenerg..

[26] Fato R., Bergamini C., Bortolus M., Maniero A.L., Leoni S., Ohnishi T. (2009). Differential effects of mitochondrial Complex I inhibitors on production of reactive oxygen species. Biochim. Biophys. Acta Bioenerg..

[27] Vinogradov A.D., Grivennikova V.G. (2016). Oxidation of NADH and ROS production by respiratory complex i. Biochim. Biophys. Acta Bioenerg..

[28] Selivanov V.A., Votyakova T.V., Pivtoraiko V.N., Zeak J., Sukhomlin T., Trucco M. (2011). Reactive oxygen species production by forward and reverse electron fluxes in the mitochondrial respiratory chain. PLoS Comput. Biol..

[29] Grivennikova V.G., Vinogradov A.D. (2006). Generation of superoxide by the mitochondrial Complex I. Biochim. Biophys. Acta Bioenerg..

[30] Grivennikova V.G., Kozlovsky V.S., Vinogradov A.D. (2017). Respiratory complex II: ROS production and the kinetics of ubiquinone reduction. Biochim. Biophys. Acta Bioenerg..

[31] Mena N.P., Bulteau A.L., Salazar J., Hirsch E.C., Núñez M.T. (2011). Effect of mitochondrial complex I inhibition on Fe-S cluster protein activity. Biochem. Biophys. Res. Commun..

[32] Beinert H., Kennedy M.C., Stout D. (1996). Aconitase as iron−sulfur protein, enzyme, and iron-regulatorypProtein. Chem. Rev..

[33] Gardner P.R., Fridovichs I. (1992). Inactivation-Reactivation of Aconitase in Escherichia coli. A sensitive measure of superoxide radical. J. Biol. Chem..

[34] Isenberg J.S., Klaunig J.E. (2000). Role of the mitochondrial membrane permeability transition (MPT) in rotenone-induced apoptosis in liver cells. Toxicol. Sci..

[35] Lindahl P.E., Öberg K.E. (1961). The effect of rotenone on respiration and its point of attack. Exp. Cell Res..

[36] Pereira C.S., Teixeira M.H., Russell D.A., Hirst J., Arantes G.M. (2023). Mechanism of rotenone binding to respiratory complex I depends on ligand flexibility. Sci. Rep..

[37] Löffler M., Jöckel J., Schuster G., Becker C. (1997). Dihydroorotat-ubiquinone oxidoreductase links mitochondria in the biosynthesis of pyrimidine nucleotides. Mol. Cell Biochem.

[38] Zhang J., Frerman F.E., Kim J.J.P. (2006). Structure of electron transfer flavoprotein-ubiquinone oxidoreductase and electron transfer to the mitochondrial ubiquinone pool. Proc. Natl. Acad. Sci. U. S. A..

[39] Green D.E. (1936). α-Glycerophosphate dehydrogenase. Biochem. J..

[40] Koza R.A., Kozak U.C., Brown L.J., Leiter E.H., MacDonald M.J., Kozak L.P. (1996). Sequence and tissue-dependent RNA expression of mouse FAD-linked glycerol-3-phosphate dehydrogenase. Arch. Biochem. Biophys..

[41] Yadav A., Alnakhli A., Vemana H.P., Bhutkar S., Muth A., Dukhande V.V. (2022). Repurposing an Antiepileptic Drug for the Treatment of Glioblastoma. Pharm. Res..

[42] Khan F., Lin Y., Ali H., Pang L., Dunterman M., Hsu W.H. (2024). Lactate dehydrogenase A regulates tumor-macrophage symbiosis to promote glioblastoma progression. Nat. Commun..

[43] Chen H., Li Y., Li H., Chen X., Fu H., Mao D. (2024). NBS1 lactylation is required for efficient DNA repair and chemotherapy resistance. Nature.

[44] Guyon J., Andrique L., Pujol N., Røsland G.V., Recher G., Bikfalvi A. (2020). A 3D Spheroid Model for Glioblastoma. J. Vis. Exp..

[45] Laemmli U.K. (1970). Cleavage of structural proteins during the assembly of the head of bacteriophage T4. Nature.

[46] Mourier A., Motori E., Brandt T., Lagouge M., Atanassov I., Galinier A. (2015). Mitofusin 2 is required to maintain mitochondrial coenzyme Q levels. J. Cell Biol..

[47] Brandt T., Mourier A., Tain L.S., Partridge L., Larsson N.G., Kühlbrandt W. (2017). Changes of mitochondrial ultrastructure and function during ageing in mice and Drosophila. Elife.

[48] Barrientos A., Fontanesi F., Díaz F. (2009). Evaluation of the mitochondrial respiratory Chain and oxidative phosphorylation system using polarography and spectrophotometric enzyme assays. Curr Protoc Hum Genet.

[49] Silva Ramos E., Larsson N.G., Mourier A. (2016). Bioenergetic roles of mitochondrial fusion. Biochim. Biophys. Acta Bioenerg..

[50] Mourier A., Matic S., Ruzzenente B., Larsson N.G., Milenkovic D. (2014). The respiratory Chain supercomplex organization is independent of COX7A2L isoforms. Cell Metab..

[51] Krumschnabel G., Fontana-Ayoub M., Sumbalova Z., Heidler J., Gauper K., Fasching M. (2015). Simultaneous high-resolution measurement of mitochondrial respiration and hydrogen peroxide production. Methods Mol. Biol..

[52] Pinson B., Moenner M., Saint-Marc C., Granger-Farbos A., Daignan-Fornier B. (2023). On-demand utilization of phosphoribosyl pyrophosphate by downstream anabolic pathways. J. Biol. Chem..

