# Supplementary material for: The antiseizure medication stiripentol inhibits mitochondrial complex I by blocking early-stage electron transfer
Source: J Biol Chem. 2026 Jan 20;302(3):111179. doi: 10.1016/j.jbc.2026.111179 (PMC13080466; doi:10.1016/j.jbc.2026.111179)
Supplement: Supplementary Material [file mmc1.pdf]

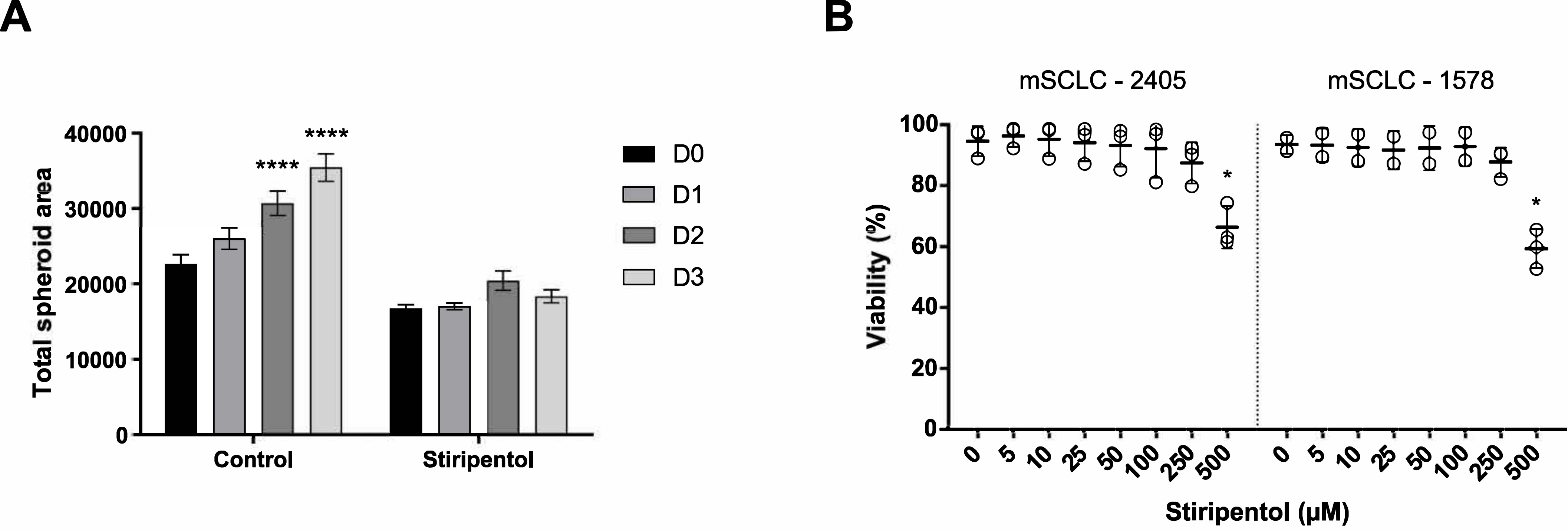

Supplementary Figure 1. Impact of stiripentol on hGB spheroids and mice cells. A, hGB spheroids growth recorded over 3 days. Data are represented as mean. Error bars represent the mean ± SEM (n=3). (8-10 spheroids per condition, n=3). **B,** Flow cytometric analysis of cell death in mSCLC 2405 (left) and mSCLC 1578 (right) cell lines. Cells were left untreated or treated with increasing concentrations of stiripentol, before analysis of apoptosis and necrosis by simultaneous detection of cell surface annexin V and DNA binding of propidium iodide. (n=3).

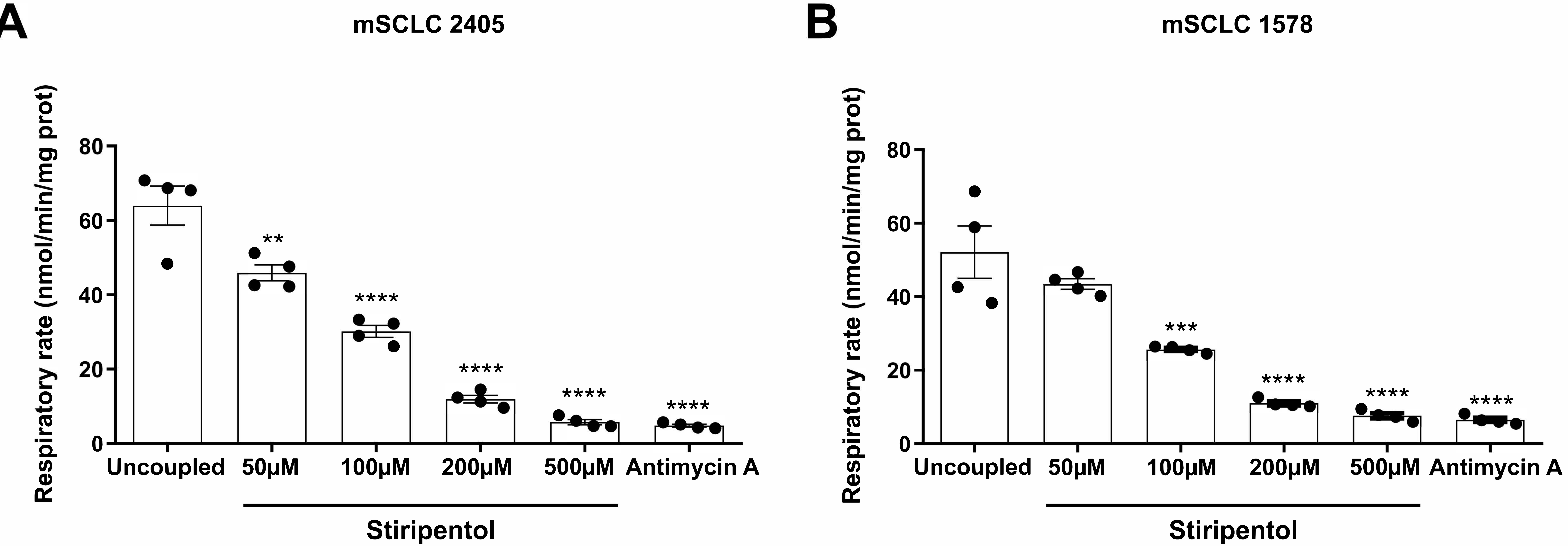

Supplementary Figure 2. Impact of stiripentol on the respiratory rate at different concentrations. A and B, Respiration of mSCLC 2405 or 1578 cells assessed under uncoupled respiratory cultured in RPMI 1640 medium with increasing concentration of stiripentol. for all experiments: **, $p < 0.01$; ***, $p < 0.001$; ****, $p < 0.0001$.

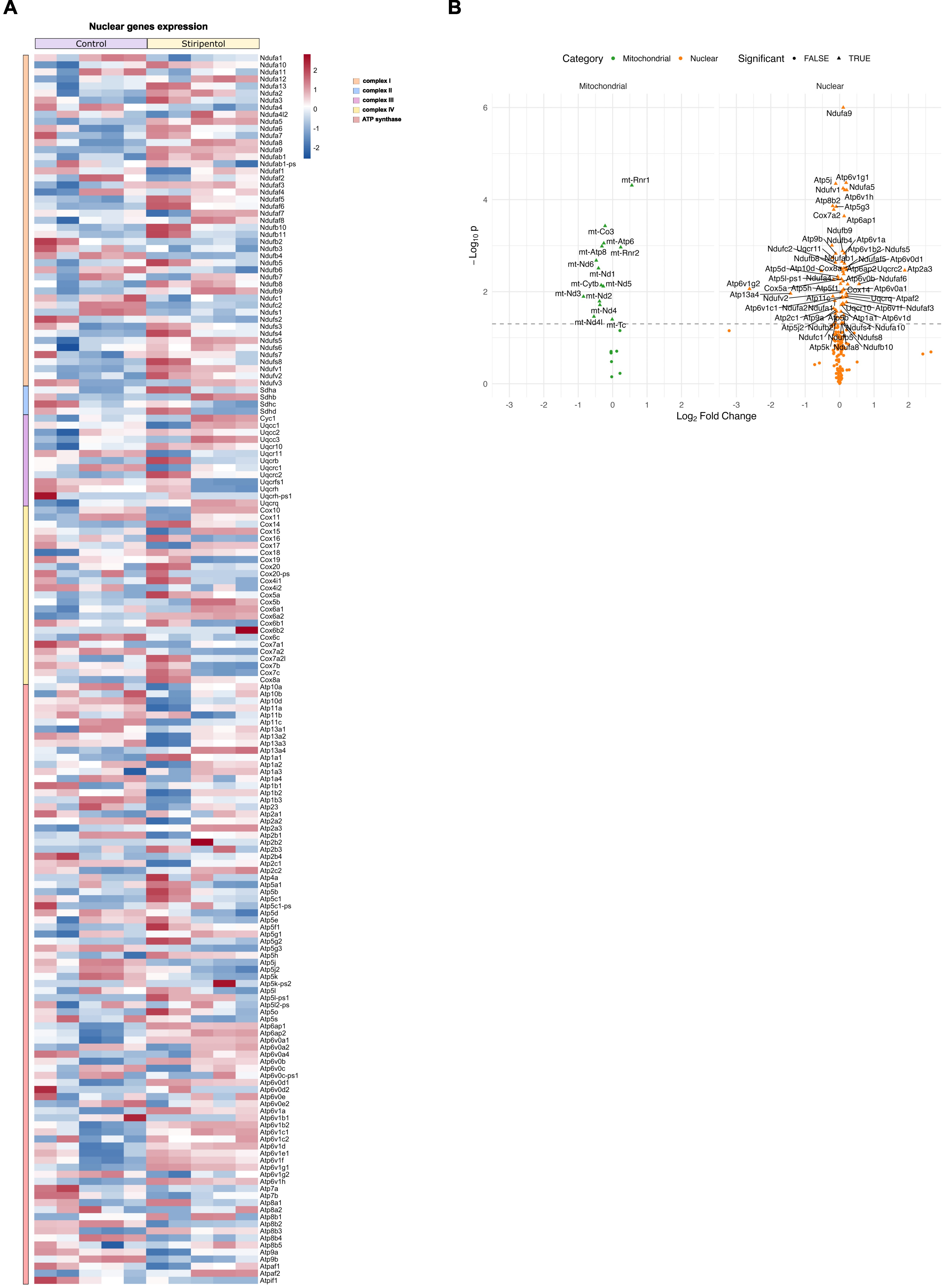

Supplementary Figure 3. Impact of stiripentol in the OXPHOS-related-nuclear-gene-expression. A, Heat map of RNA sequencing for OXPHOS-related-nGenes in mSCLC 2405 with or without 250µM stiripentol (n=5). **B,** Volcano plot of RNA sequencing for OXPHOS-related-mitochondrial and nuclear-genes in mSCLC 2405 with or without 250µM stiripentol. the significativity is esthablished for pValue < 0.05 (n=5).

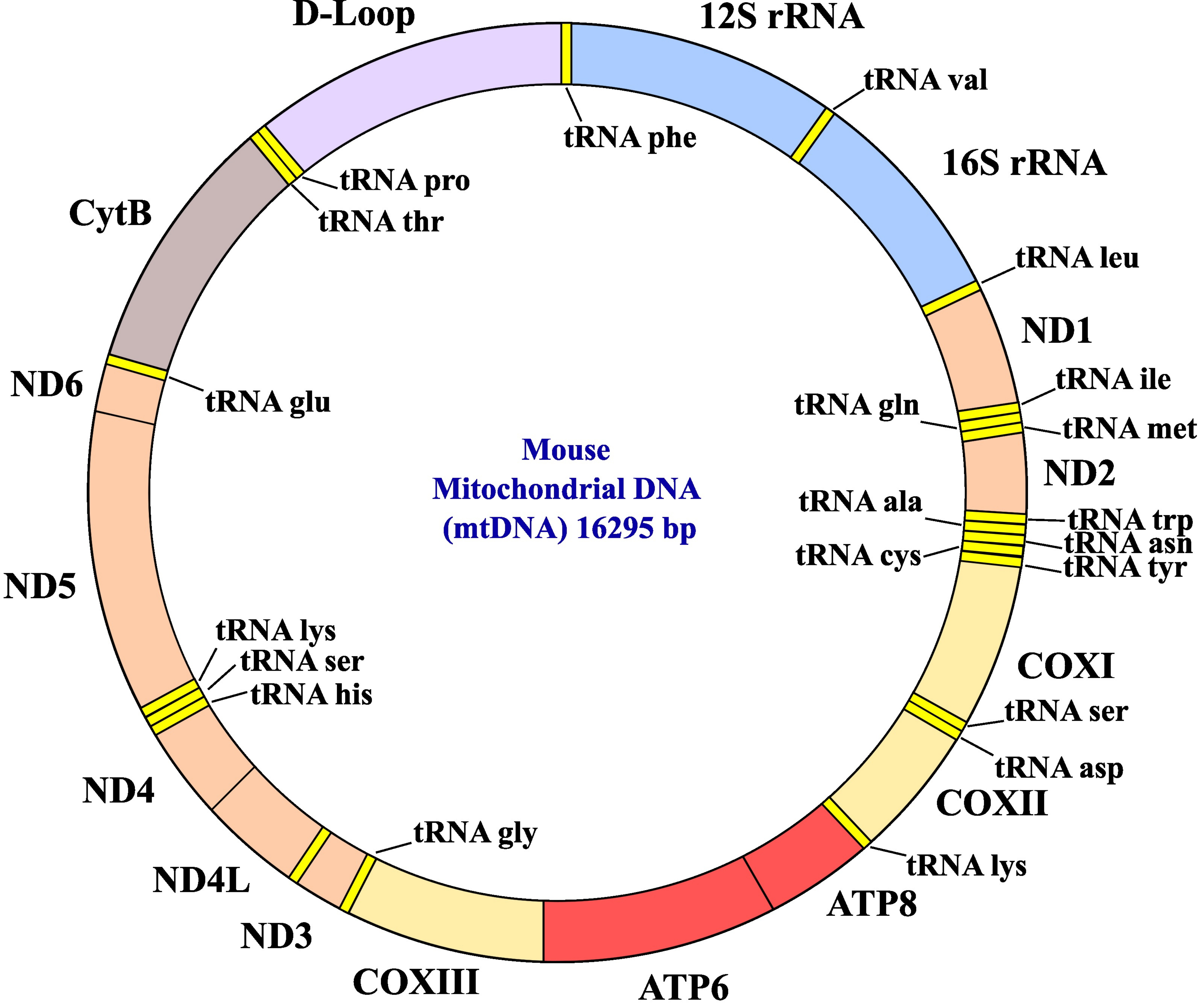

Supplementary Figure 4. Map of the mouse mitochondrial genome. 12S rRNA and 16S rRNA are coding by Rnr1 and Rnr2 respectively. tRNA leu is coding by Tl1, tRNA pro is coding by Tp, tRNA gl is coding by Tq, tRNA ser is coding by Ts1, tRNA asn is coding by Tn, tRNA cys is coding by Tc, tRNA tyr is coding by Ty.

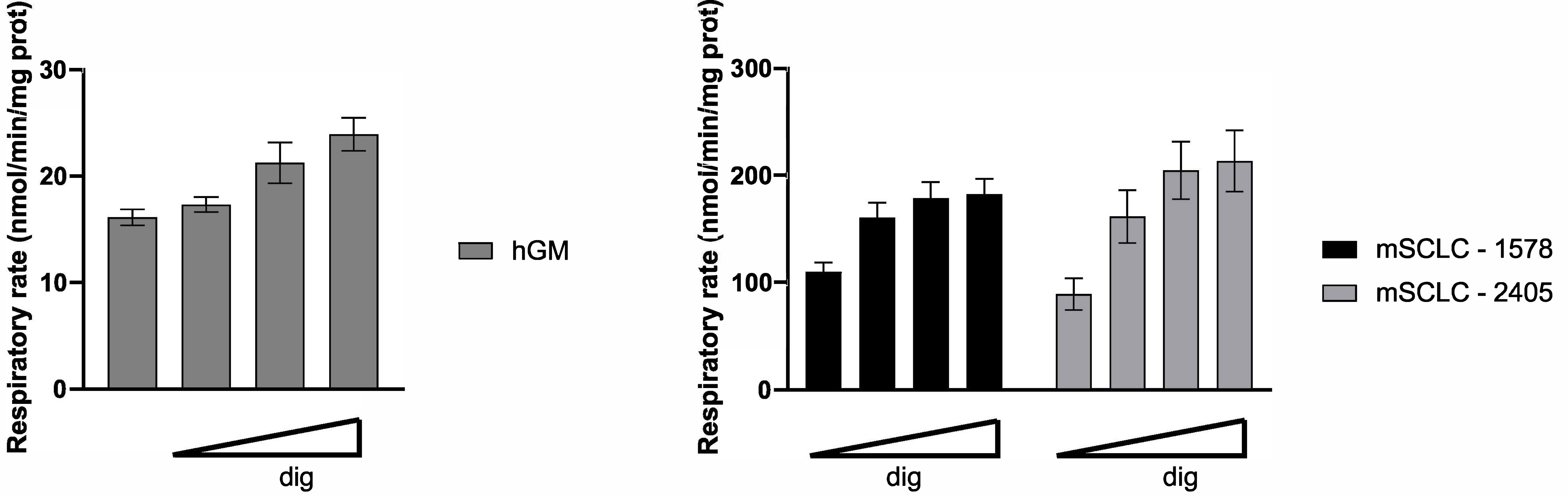

Supplementary Figure 5. Impact of digitonine on the respiratory rate at different concentrations. A and B, Respiration of hGB (A) or mSCLC 2405 or 1578 (B) cells assessed in respiratory buffer with increasing concentration of stiripentol. for all experiments: *, $p < 0.05$, **, $p < 0.01$.

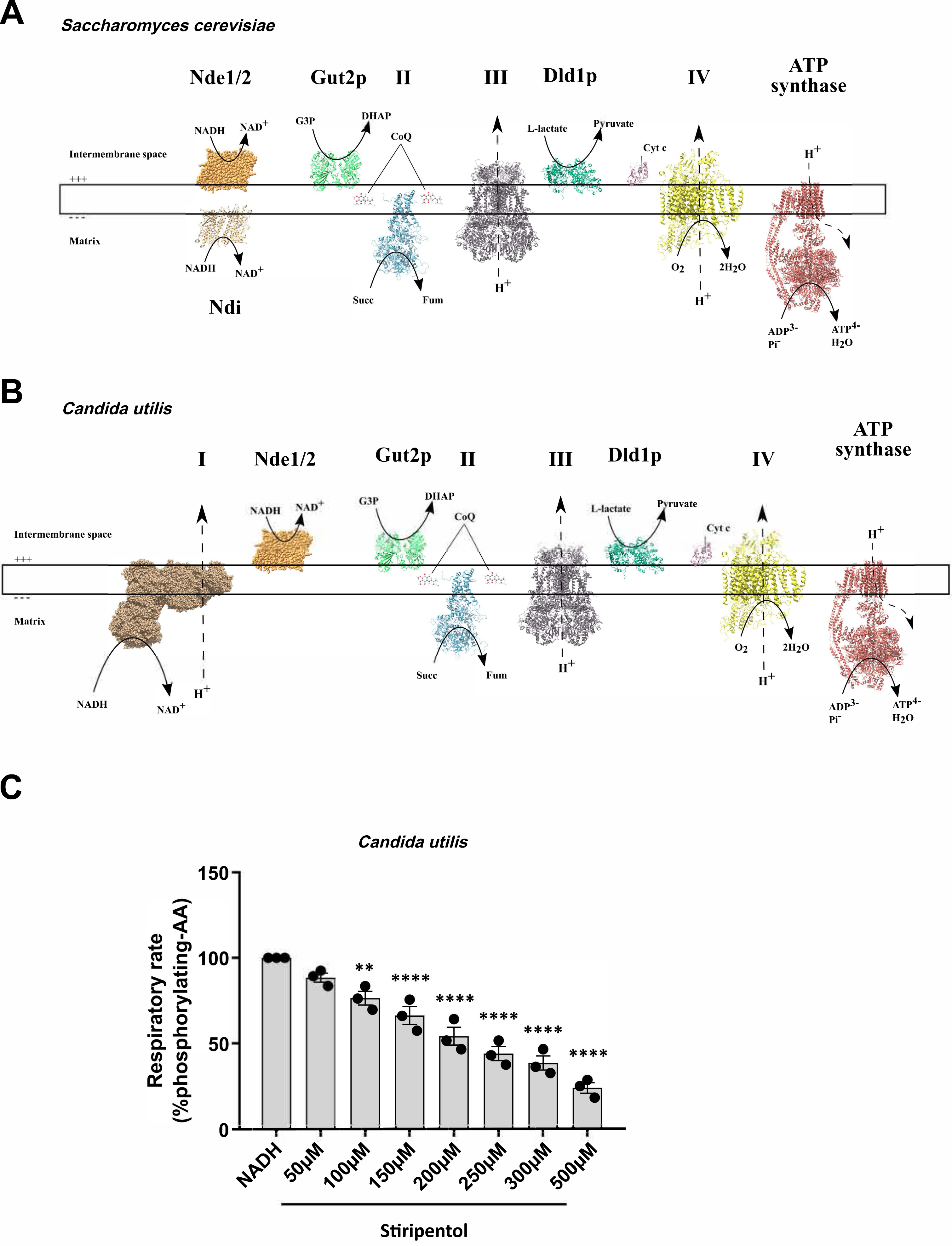

Supplementary Figure 6. Respiratory chain of different yeast strain. A, B, Oxidative phosphorylation system in yeast *Saccharomyces cerevisiae* (A) and *Candida utilis* (B). In *S. cerevisiae*, the respiratory chain (RC) has two types of NADH dehydrogenases, one internal (Ndi) and one external (Nde). *C. utilis* also has Nde however its Ndi is coupled to proton extrusion has a mammalian complex I. The following PDB structures were used for this figure: (A) Ndi: PDB: 4G9K from *S. cerevisiae*; (A) and (B) Nde1/2: PDB: 4G6H (NDH with NADH from *S. cerevisiae*); Complex II: PDB: 1ORZ from *S. cerevisiae*; Gut2p: PDB: 3PYM from *S. cerevisiae*; Complex III: PDB: 1KB9 from *S. cerevisiae* Complex IV: PDB: 6YMY from *S. cerevisiae;* Cyt c: PDB: 1CRH from *S.cerevisiae*; L-LDH: PDB: 1LDC from *S. cerevisiae*; ATPsynthase: PDB: 6O7V from *S. cerevisiae*; ANT: PDB: 4C9G from *S. cerevisiae*; (B) Complex I : PDB: 7O71 from *Yarrowia lipolytica*. **C,** oxygen consumption assessed with permeabilised *C. utilis* mitochondrial fed with NADH and increased concentrations of stiripentol. Results are expressed as a percentage of NADH condition for each experiment. The bar graph shows the mean percentage of NADH condition ± SEM (n=4). for all experiments: **, $p < 0.01$; ****, $p < 0.0001$.
